# Stable carbon and nitrogen isotopes identify nuanced dietary changes from the Bronze and Iron Ages on the Great Hungarian Plain

**DOI:** 10.1038/s41598-022-21138-y

**Published:** 2022-10-10

**Authors:** Ashley McCall, Beatriz Gamarra, Kellie Sara Duffett Carlson, Zsolt Bernert, Andrea Cséki, Piroska Csengeri, László Domboróczki, Anna Endrődi, Magdolna Hellebrandt, Antónia Horváth, Ágnes Király, Krisztián Kiss, Judit Koós, Péter Kovács, Kitti Köhler, László Szolnoki, Zsuzsanna K. Zoffmann, Kendra Sirak, Tamás Szeniczey, János Dani, Tamás Hajdu, Ron Pinhasi

**Affiliations:** 1 Dublin, Ireland; 2 Institut Català de Paleoecologia Humana i Evolució Social (IPHES), Zona Educacional 4, Campus Sescelades URV (Edifici W3), 43007 Tarragona, Spain; 3 Departament d’Història i Història de l’Art, Universitat Rovira i Virgili, Avinguda de Catalunya 35, 43002 Tarragona, Spain; 4 Department of Evolutionary Anthropology, University of Vienna, 1030 Vienna, Austria; 5 Human Evolution and Archaeological Sciences, University of Vienna, 1030 Vienna, Austria; 6 Department of Anthropology, Hungarian Natural History Museum, Ludovika tér 1-3, 1083 Budapest, Hungary; 7 Archeodata 1998 Ltd., Polgár, Hungary; 8 Department of Archaeology, Herman Ottó Museum, Görgey Artúr u. 28, 3529 Miskolc, Hungary; 9 Department of Archaeology, Dobó István Castle Museum, Vár 1, Eger, 3300 Hungary; 10 Department of Prehistoric and Migration Period, Budapest History Museum, Aquincum Museum and Archaeological Park, Szentendrei út 135, 1031 Budapest, Hungary; 11 Institute of Archaeology, Research Centre for Humanities, Hungarian Academy of Sciences, Tóth Kálmán utca 4, 1097 Budapest, Hungary; 12 Department of Biological Anthropology, Institute of Biology, Faculty of Science, Eötvös Loránd University, Pázmány Péter sétány 1/c, 1117 Budapest, Hungary; 13 Damjanich János Museum, Kossuth tér 4, 5000 Szolnok, Hungary; 14 Déri Museum, Déri tér 1, 4026 Debrecen, Hungary; 15 Department of Anthropology, Hungarian National Museum, Múzeum krt. 14-16, Budapest, 1083 Hungary; 16 Department of Genetics, Harvard Medical School, Boston, MA 02115 USA; 17 Department of Human Evolutionary Biology, Harvard University, Cambridge, MA USA

**Keywords:** Anthropology, Archaeology

## Abstract

The Great Hungarian Plain (GHP) served as a geographic funnel for population mobility throughout prehistory. Genomic and isotopic research demonstrates non-linear genetic turnover and technological shifts between the Copper and Iron Ages of the GHP, which influenced the dietary strategies of numerous cultures that intermixed and overlapped through time. Given the complexities of these prehistoric cultural and demographic processes, this study aims to identify and elucidate diachronic and culture-specific dietary signatures. We report on stable carbon and nitrogen isotope ratios from 74 individuals from nineteen sites in the GHP dating to a ~ 3000-year time span between the Early Bronze and Early Iron Ages. The samples broadly indicate a terrestrial C_3_ diet with nuanced differences amongst populations and through time, suggesting exogenous influences that manifested in subsistence strategies. Slightly elevated δ^15^N values for Bronze Age samples imply higher reliance on protein than in the Iron Age. Interestingly, the Füzesabony have carbon values typical of C_4_ vegetation indicating millet consumption, or that of a grain with comparable δ^13^C ratios, which corroborates evidence from outside the GHP for its early cultivation during the Middle Bronze Age. Finally, our results also suggest locally diverse subsistence economies for GHP Scythians.

## Introduction

**Figure 1:**
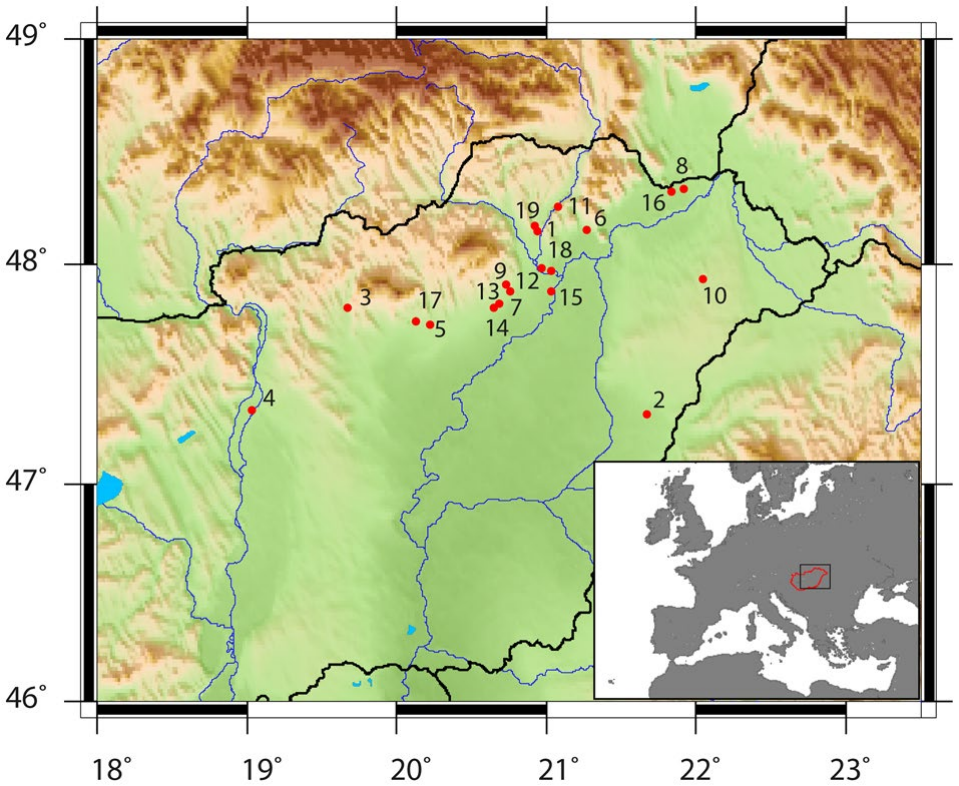
Map showing the location of sites. 1. Ongaújfalu-Állami gazdaság, 2. Konyár-Pocsaji műút, 3. Apc-Berekalja I, 4. Szigetszentmiklós-Üdülősor, 5. Kompolt-Kígyósér, 6. Mezőzombor-Községi temető, 7. Mezőkeresztes-Csincse-tanya, 8. Nagyrozvágy-Pap-domb, 9. Vatta-Dobogó, 10. Ófehértó-Almezői dűlő, 11. Felsődobsza site 2, 12. Köröm-Kápolnadomb, 13. Mezőkeresztes, 14. Mezőkeresztes-Cet halom M3-10, 15. Oszlár-Nyárfaszög, 16. Pácin-Alsókenderszer, 17. Ludas-Varjú-dűlő, 18. Kesznyéten-Szérűskert, 19. Szikszó-Hell Ring. Generic Mapping Tools 4.5.13^71^ and the topographic ETOPO dataset^72^ were used to create this map.

Located centrally and comprising the majority of the Carpathian Basin, the Great Hungarian Plain (GHP) forms a lowland confluence connecting the Balkans, Pontic Steppe, and Central Europe^1,2^. The GHP acted as a geographic funnel for population movement whereby new people and their ideas and ways of life, including subsistence strategies, migrated through Europe. As such, it functioned as a region of major cultural and technological transition throughout prehistory^3,4^. It is thus a crucial region for identifying and investigating dietary trends between prehistoric time periods and cultures. We report on carbon and nitrogen stable isotope values from 74 individuals from nineteen sites in the GHP (Fig. 1) dating to a ~ 3000-year transect between the Early Bronze and Early Iron Ages (Table 1). Specifically, we address two main questions: (1) Can nuanced dietary changes be detected across millennia? (2) If changes are detected, what do they imply regarding prehistoric trans-Carpathian cultural communication and trade?

**Table 1:** Summary of the prehistoric time periods and their associated cultures and subsistence practices in the GHP.

Time period	Date range	Associated sampled cultures	Subsistence practices
Early Bronze Age	2600 to 2000/1900 BCE	Nyírség, Proto-Nagyrév, Bell Beaker, Hatvan	Intensive crop cultivation (barley, wheat, legumes) and animal husbandry
Middle Bronze Age	2000/1900 to 1450/1400 BCE	Füzesabony, Otomani/Ottomány	Intensive crop cultivation (barley, einkorn, emmer, legumes, rye, legumes) and animal husbandry
Late Bronze Age	1450/1400 to 800/900 BCE	Piliny/Kyjatice, pre-Gáva, Gáva	Intensive crop cultivation (einkorn, emmer, barley, legumes); common millet as staple crop
Early Iron Age	800/900 to 650 BCE	Pre-Scythian (Mezőcsát), Scythian (Vekerzug)	Pastoral/semi-nomadism/transhuman pastoralism; stockbreeding; crop cultivation
Middle Iron Age	650 to 450 BCE	Scythian (Vekerzug)	Pastoral/semi-nomadism/transhuman pastoralism; stockbreeding; crop cultivation

Adapted from Gamarra et al.^58^.

### Stable Isotopes

The application of stable isotopes to prehistoric dietary analyses is complex as many factors affect the ratios obtained from samples^5–9^. In brief, organisms incorporate carbon and nitrogen from diet material. For carbon, most plants fall under one of two categories (C_3_ and C_4_) based on their photosynthetic pathway, which makes it possible to distinguish general plant groups. C_3_ plants, which include temperate grasses and domesticated cereals from terrestrial ecosystems, exhibit carbon isotope values (^13^C/^12^C ratio compared to the standard VPDB or δ^13^C) from − 38 parts per mil (‰) to − 22‰, with a mean of − 26.5‰^10–12^. C_4_ plants, such as maize, sorghum, and millet, exhibit higher δ^13^C ratios, ranging between − 21 and − 9‰, with a mean of − 12.5‰^10,13,14^. Dietary nitrogen (^15^ N/^14^ N ratio compared to the standard AIR or δ^15^N) is incorporated via protein at a stepwise factor of about + 3 to 5‰^15–17^. Plants in some terrestrial ecosystems can range between − 15 and − 10‰^18^; however, aquatic resources—including freshwater—can exhibit comparatively ^15^ N-enriched values due to the relative complexity of the foodweb^19,20^. Moreover, both nitrogen and carbon isotope ratios are affected by climate^21–23^, soil conditions^24,25^, elevation^26^, water stress^27,28^, health of the individual^29–31^, and breastfeeding^8^. Lastly, milk consumption, not unlike breastfeeding, augments δ^15^N values much like that seen in other dietary trophic level increases^32^.

### Cultural and dietary context

Animal husbandry, primarily of cattle, was the predominant subsistence practice during the Middle Copper Age of people migrating to the Carpathian Basin from the Eastern Steppe^33–35^. The contemporaneous cultures of the Late Copper Age, including the Baden, Vučedol, and Coţofeni, continued the tradition of animal husbandry and land cultivation^35–37^. A transformation from the ‘monolithic’ Baden culture to more varied and smaller regional Bronze Age communities was shaped either by internal developments or foreign influences, including population movement, of, for example, the Yamnaya, who arrived from the east during the Transitional Period (~ 2800 to 2600 BCE)^38–40^. This was followed by the expansion of the Bell Beaker who migrated from the west (~ 2500 BCE)^38,41^, and was accompanied by several independent cultures (e.g., Makó-Kosihy-Caka, Nagyrév, Somogyvár-Vinkovici), all practicing intensive cereal cultivation and animal husbandry^42^. However, only small groups settled along Danube River routes^38–40^.

The transition from the Early Bronze to Middle Bronze Age is marked by the development of the more sedentary Hatvan, Otomani/Ottomány, and Füzesabony cultures, all of whom occupied tells^36,37,39,41,43–45^. These groups and others coexisted for centuries, although not necessarily peacefully, until the end of the Middle Bronze Age^37,46^. The Füzesabony were partly contemporaneous with and subsequent to the Hatvan, with no indications that upon their arrival they usurped the former culture^45^. The Otomani-Füzesabony is associated with increased socio-political and metallurgical complexity in the Carpathian Basin, as evidenced by tell sites, communal cemeteries, and advanced trade networks^47^. By this point the plow had been introduced^48^, with communities cultivating cereals like wheat and barley, vegetables and fruits, and likely fodder crops to feed cattle, pig, goat, sheep, and horses^36,49^.

Although there was profound cultural diversification during the Early Bronze and Middle Bronze Ages, by the Late Bronze Age cultures appear to homogenize over large geographic regions, much like that which occurred between the Late Neolithic and Early Copper Age, as manifested in the reduction of local cultural expression. The emergence of several cultures, including the Piliny/Kyjatice in the northern mountain range, and Gáva east of the Tisza, likely resulted from interregional contacts between groups occupying different ecological zones, resulting in increased trade and information flow^50^. This is further supported by the spread and increased cultivation of millet^51,52^.

Late Bronze Age villages were seemingly abandoned, and new traditions and material culture appeared in the eastern parts of the Carpathian Basin at the beginning of the Iron Age (~ 900/800 BCE), namely on the central and southern part of the GHP, in the Northern Mountain Range, and in Transylvania. The Early Iron Age of the GHP is largely underrepresented in the archaeological record, perhaps because the cultures of this period, in particular the pre-Scythian (Mezőcsát), who mainly occupied the central and northern parts of the GHP^53,54^, were nomadic stockbreeders of gregarious animals (e.g., cattle, sheep, horse), unlike their more sedentary predecessors^53,55^. The Scythian (Vekerzug in the GHP) culture subsequently emerged and continued into the Middle Iron Age. Excavations of Vekerzug settlements indicate that agriculture and animal husbandry were practised along with highly developed iron metallurgy and ceramic manufacture^53,56^. Various other Middle Iron Age cultures occupied this region until the end of the fifth century BCE, when the Celts began their conquests and interrupted development of local cultures, not just in the Tisza region, but throughout the Carpathian Basin^53,57^. The associated cultures in the present dataset, and their associated dietary information, can be found in Table 1.

### Previous archaeochemistry of the Great Hungarian Plain

To assess links between diet and cultural evolution on the GHP, stable isotope and ancient DNA (aDNA) research has been conducted on samples from the Neolithic through Iron Age^42,58–61^. Previous carbon and nitrogen stable isotope analyses of human and faunal osteological samples from this region have focused primarily on Neolithic and Copper Age populations, reporting a transformation in subsistence strategies during the Late Neolithic and Copper Age towards increased consumption of animal protein compared to the previous subperiods^58,62–65^. Gamba et al.^59^ analysed the genomes of thirteen GHP individuals dating to between the Early Neolithic and Early Iron Age; the Bronze and Iron Age samples provided evidence for genomic turnover that contrasted the genetic continuity observed during the Neolithic and Copper Age. Allentoft et al.’s^42^ study of Eurasian genomes reported dynamic migrations during the Bronze Age, as well as the rise of the allele that confers the lactase gene, while de Barros Damgaard et al.^66^ found that Scythian groups were genetically comprised of Late Bronze Age herders, farmers, and hunter-gatherers. Comparing carbon and nitrogen isotopic ratios with aDNA results from GHP samples, Gamarra et al.^58^ found no associations between dietary, cultural, and genetic shifts from the Early Neolithic to Iron Age; however, Bronze and Iron Age individuals exhibited a diet higher in C_4_ plants, such as millet, which is a typical agricultural crop at this time in this region, compared to those from the Neolithic and Copper Age.

Genetic turnover, and technological evolution (e.g., changes in metallurgy during the Bronze Age: copper smelting to make Bronze, new casting techniques^67,68^, sheet metal manufacture^69^; and in the Iron Age: the ability to more locally produce iron, which affected political economies and the production of tools)^70^ were thus non-linear, influencing the dietary strategies of numerous cultures that intermixed and overlapped through time. Owing to the complexities of these prehistoric cultural and demographic processes, the present study thus aims to improve our understanding of diachronic and culture-specific dietary signatures as revealed by the archaeology and stable isotopes both between and within chronological periods and cultures.

## Results

**Figure 2:**
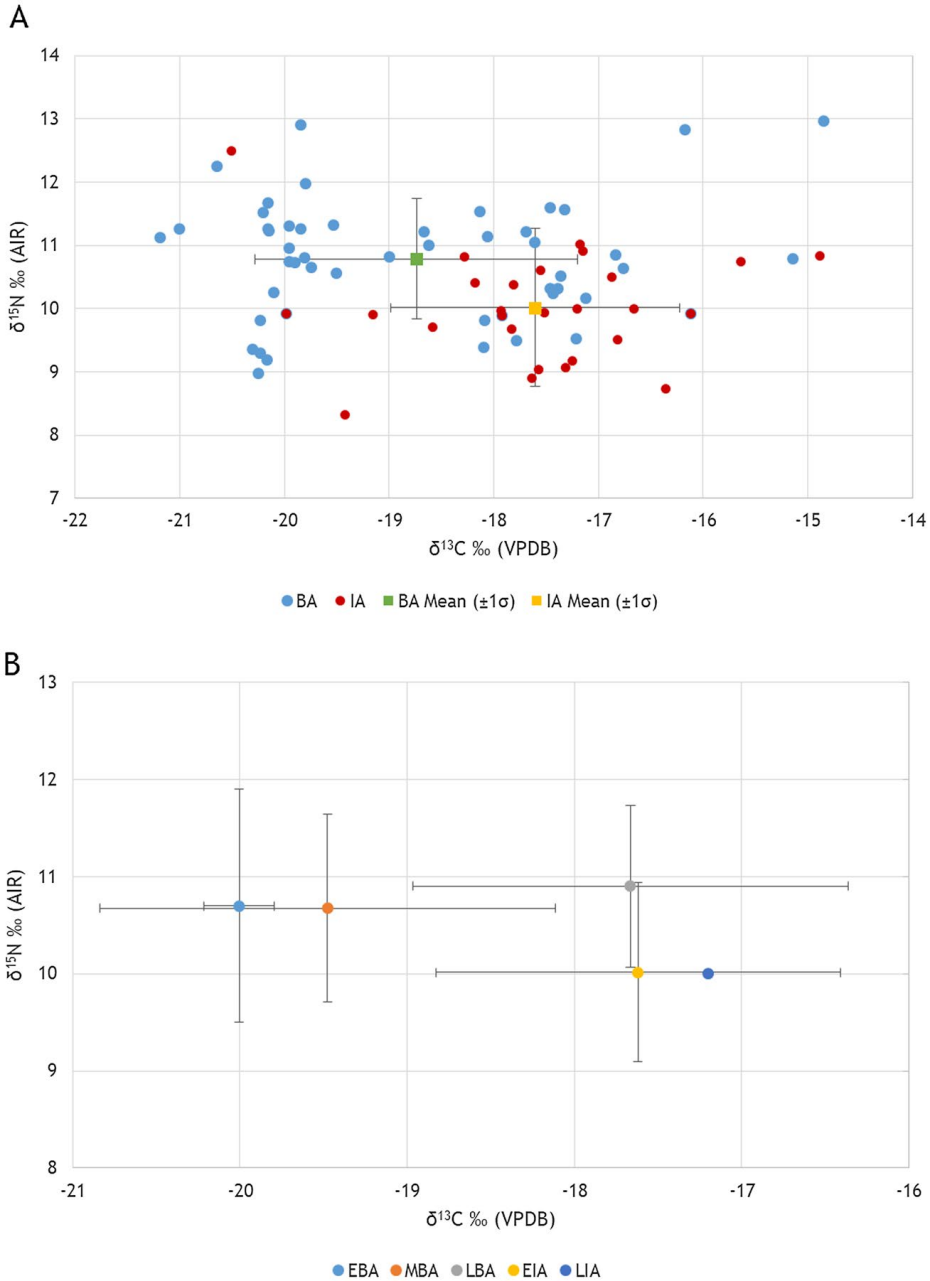
Scatterplots of human δ^13^C and δ^15^N ratios with mean δ^13^C and δ^15^N ratios (± 1 σ) by period (**A**) Bronze Age/BA (n = 50) and Iron Age/IA (n = 24) and subperiod (**B**) Early Bronze Age/EBA (n = 8), Middle Bronze Age/MBA (n = 18), Late Bronze Age/LBA (n = 22), and Early Iron Age/EIA (n = 24).

Archaeological and burial information are found in Supplementary Material S1, isotopic ratios, palaeodemographic information, and quality criteria are provided for each sample in Supplementary Material S2, and statistical tables are listed in Supplementary Material S3. Figure 2 illustrates the range of the stable carbon and nitrogen isotopic results of samples from this study coupled with data of Gamarra et al.^58^ and McCall^73^. The range of δ^13^C ratios for the entire dataset is − 21.2 to − 14.8‰ (mean = −18.3‰ ± 1.6‰ (1σ)); the δ^15^N value range for the entire dataset is + 8.3 to 12.9‰ (mean =  + 10.5‰ ± 0.9‰ (1σ); Table 2). Overall, most samples indicate a terrestrial C_3_ diet with nuanced statistical differences between certain groups, suggesting external influences that manifested in the diet. This is in keeping with what is known about food practices at the time, and is also congruent with previous isotopic analyses^34,58^.

**Table 2:** Summary of human δ^13^C and δ^15^N results of sample by period and subperiod with ‰ range, mean, and SD (± 1σ).

Period/subperiod	n	δ^13^C range (‰)	Mean	SD	δ^15^N range (‰)	Mean	SD
Bronze Age	50	− 21.2 to − 14.8	− 18.7	1.5	8.9 to 12.9	10.7	0.9
Iron Age	24	− 20.5 to − 14.9	− 17.5	1.2	8.3 to 12.4	10.0	0.9
Early Bronze Age	8	− 20.3 to − 19.7	− 20.0	0.2	9.3 to 12.9	10.7	1.2
Middle Bronze Age	18	− 21.1 to − 16.7	− 19.4	1.3	8.9 to 12.2	10.7	0.9
Late Bronze Age	22	− 19.9 to − 14.8	− 17.7	1.3	9.8 to 12.9	10.9	0.8
Early Iron Age	24	− 20.5 to − 14.8	− 17.5	1.2	8.3 to 12.4	10.0	0.9

### Chronological variability

**Figure 3:**
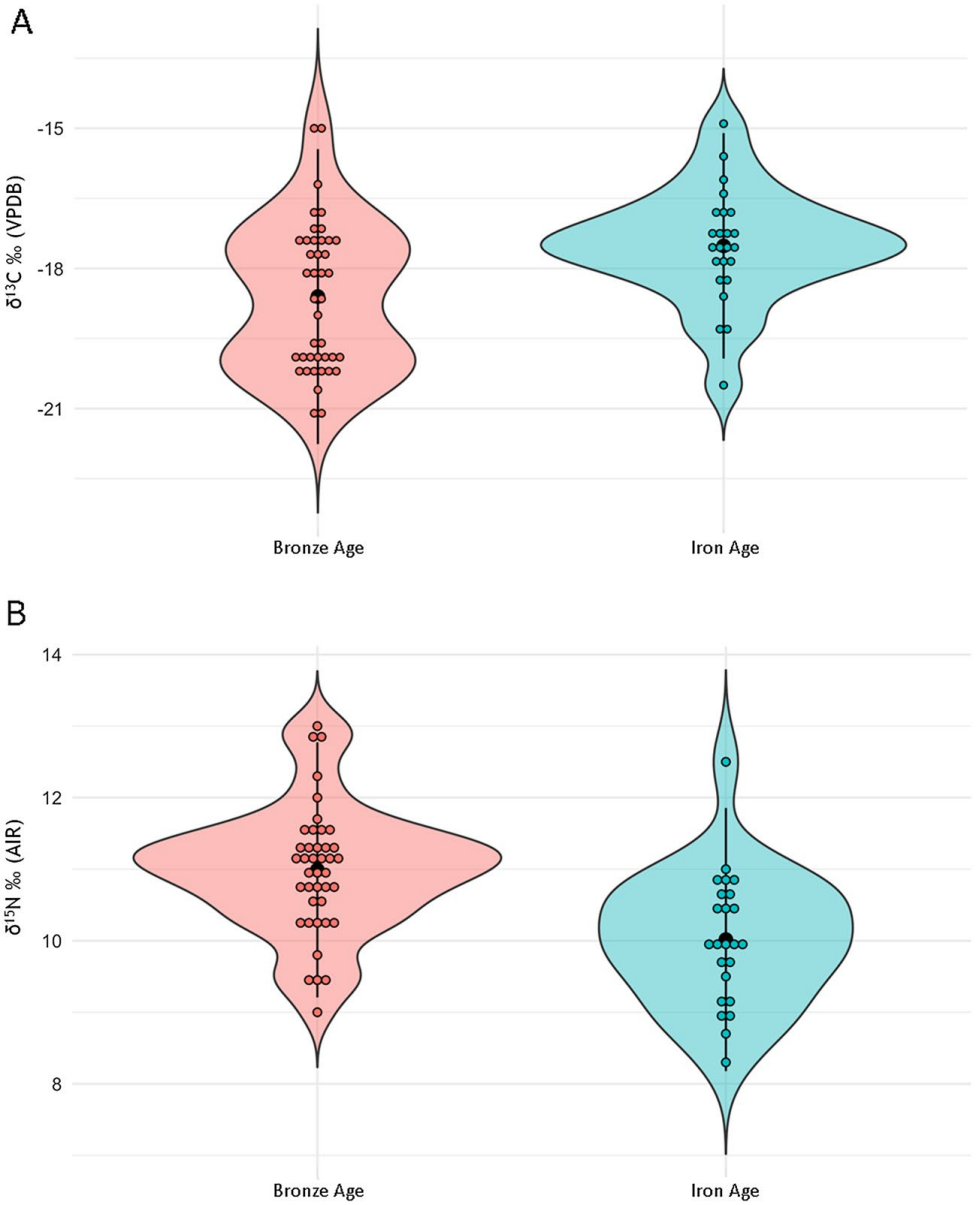
Violin plot of human (**A**) δ^13^C and (**B**) δ^15^N ratios by period: Bronze Age (n = 50) and Iron Age (n = 24). Center black dot represents mean; center black line represents distribution.

**Figure 4:**
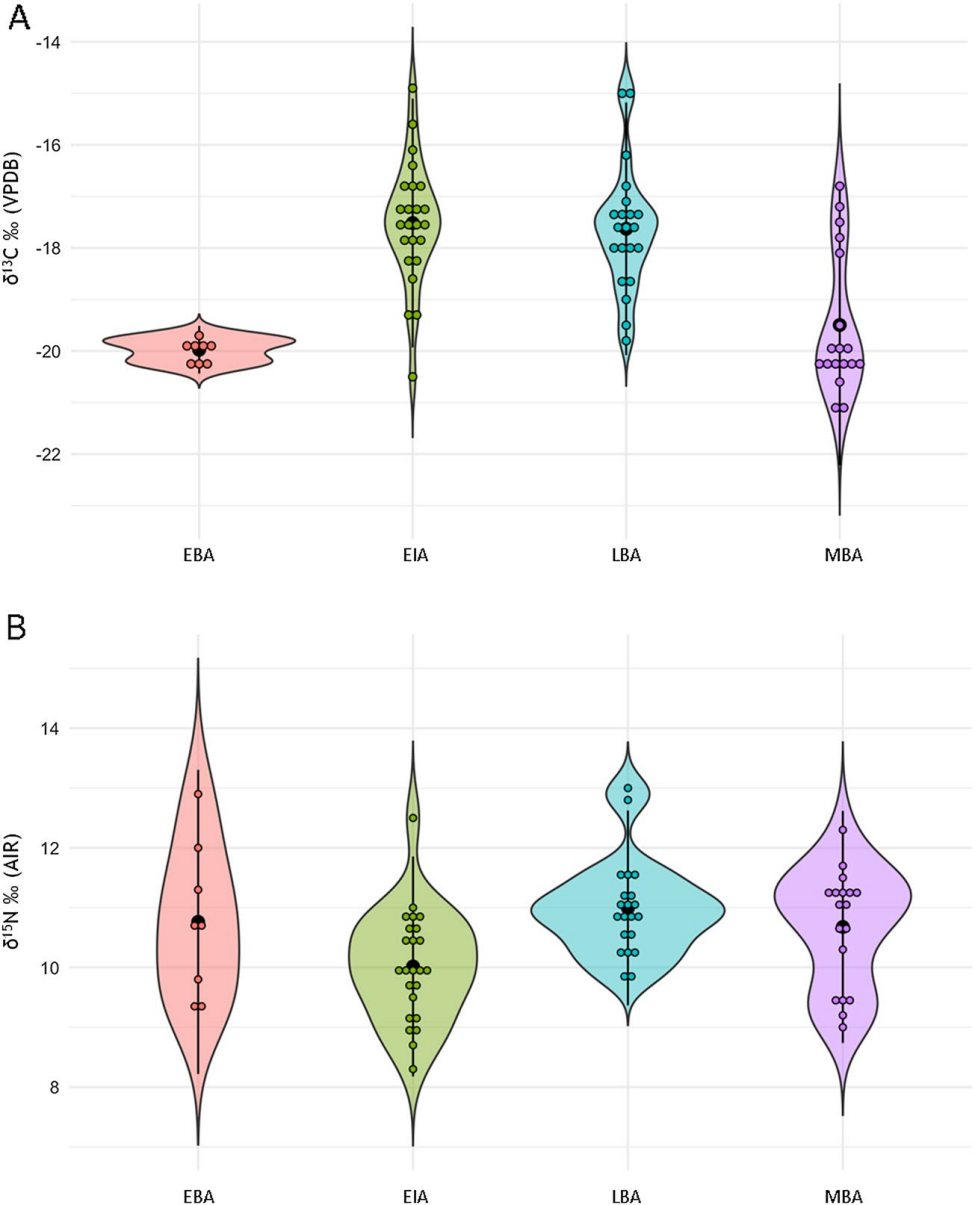
Violin plot of human (**A**) δ^13^C and (**B**) δ^15^N ratios by subperiod: Early Bronze Age (n = 8), Middle Bronze Age (n = 18), Late Bronze Age (n = 22), and Early Iron Age (n = 24). Center black dot represents mean; center black line represents distribution.

The δ^13^C and δ^15^N results per period and subperiod are summarized in Table 2; tests of normality are found in Supplementary Material S3a, S3b, S3c, and S3d. There is an overall statistical difference for δ^13^C values between the Bronze and Iron Ages (Mann–Whitney U: U = 900.5; p = 0.001; Fig. 3A; Supplementary Material S3e); the Early Bronze, Middle Bronze, Late Bronze, and Early Iron Ages also exhibit significant statistical differences (Kruskal–Wallis: X^2^ = 31.122, p < 0.001; Fig. 4A; Supplementary Material S3f). As the Kruskal–Wallis test (p < 0.001) indicated differences between the four subperiods analyzed, pairwise Mann–Whitney tests were employed, revealing a significant difference between the Early Bronze and Middle Bronze Ages compared with the Late Bronze and Early Iron Ages (Supplementary Material S3g).

Similarly, for δ^15^N ratios there is an overall statistical difference between the Bronze and Iron Ages (independent samples t-test t_73_ = 3.369, p = 0.001; Fig. 3B; Supplementary Material S3h). The Tukey’s post hoc analysis (Supplementary Material S3i), performed after a significant ANOVA (F_(3,68)_ = 4.250, p = 0.008) result (Supplementary Material S3j), identified differences between the Late Bronze and Early Iron Ages, and differences among the Early, Middle, and Late Bronze Ages (Fig. 4B). Furthermore, there is a negative correlation (− 0.418, p < 0.001) across the four subperiods when comparing mean δ^13^C and δ^15^N ratios, seen when the increase in overall δ^13^C values correlates with a decrease in δ^15^N values.

### Cultural variability

**Figure 5:**
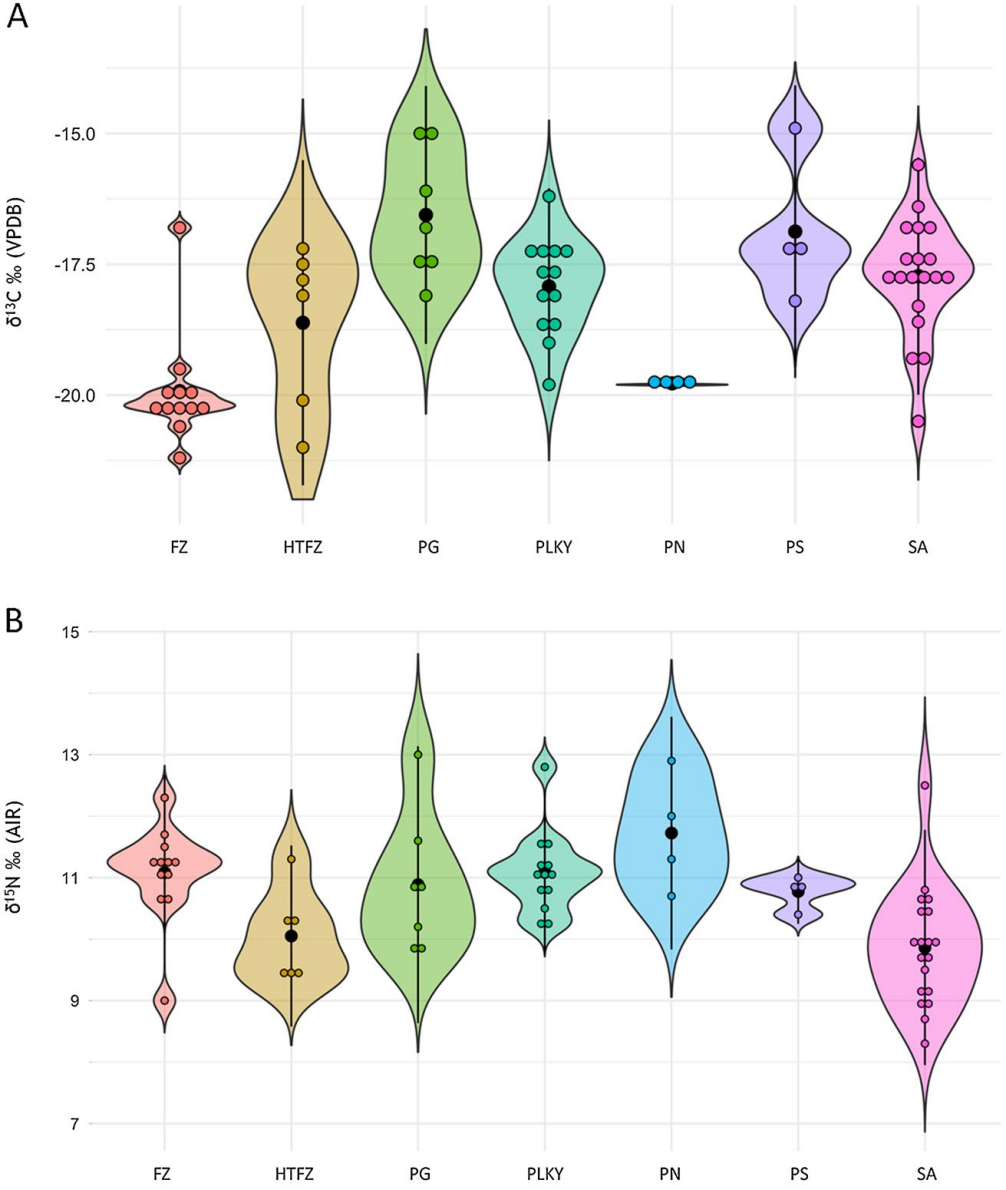
Violin plot of human δ^13^C and δ^15^N ratios by cultures listed in roughly chronological order: PN (Proto-Nagyrév, n = 4), FZ (Füzesabony, n = 12), HFTZ (Hatvan or Füzesabony, n = 6), PLKY (Piliny or Piliny/Kyjatice, n = 14), PG (pre-Gáva or Gáva, n = 8), PS (Pre-Scythian/Mezőcsát, n = 4), and SA (Scythian/Vekerzug, n = 19). Center black dot represents mean; center black line represents distribution.

The ranges for δ^13^C and δ^15^N ratios by culture are listed in Table 3; the δ^13^C and δ^15^N results are represented in Fig. 5; the tests of normality can be found in Supplementary Material S3k and S3l. For the inter-culture comparison (n = 67) there is an overall statistical difference between δ^13^C values (Kruskal–Wallis: X^2^ = 25.159, p < 0.001) with the Füzesabony found to be significantly different from the Gáva and Scythian (Fig. 5A; Supplementary Material S3m). Similarly, the Proto-Nagyrév compared with the Gáva, Piliny/Kyjatice, pre-Scythian, and Scythian also demonstrate a notable difference in δ^13^C values (Pairwise Mann–Whitney, Supplementary Material S3n). The comparison of δ^15^N ratios revealed an overall statistical difference between cultures (one-way ANOVA: F_(5,60)_ = 4.860, p = 0.001; Fig. 5B; Supplementary Material S3o). However, Scythian is the only culture with consistent differences for δ^15^N values compared with the Füzesabony, Proto-Nagyrév, and Piliny/Kyjatice cultures (Tukey’s test, Supplementary Material S3p).

**Table 3:** Summary of human δ^13^C and δ^15^N results by culture with ‰ range, mean, and SD (± 1σ).

Cultures	Time period(s)	n	δ^13^C range (‰)	Mean	SD	δ^15^N range (‰)	Mean	SD
Nyírség	Early Bronze Age	1	− 20.2	N/A	N/A	9.3	N/A	N/A
Proto-Nagyrév	Early Bronze Age	4	− 19.8 to − 19.7	− 19.8	0.1	10.6 to 12.9	11.7	0.9
Bell Beaker	Early Bronze Age	2	− 20.3 to − 20.2	− 20.2	N/A	9.3 to 9.8	9.5	N/A
Hatvan	Early Bronze Age	1	− 19.9	N/A	N/A	10.7	N/A	N/A
Hatvan or Füzesabony	Early Bronze Age/Middle Bronze Age	6	− 21.0 to − 17.2	− 18.6	1.6	9.4 to 11.3	10.0	0.7
Füzesabony	Middle Bronze Age	12	− 21.1 to − 16.7	− 19.4	1.3	8.9 to 12.2	10.7	0.9
Otomani/Ottomány	Middle Bronze Age	1	− 20.1	N/A	N/A	9.2	N/A	N/A
Piliny or Piliny/Kyjatice	Late Bronze Age	14	− 19.8 to − 16.1	− 18.0	1.0	10.1 to 12.8	11.1	0.6
pre-Gáva or Gáva	Late Bronze Age, Early Iron Age	8	− 18.0 to − 14.8	− 16.7	1.2	9.8 to 12.9	10.7	1.0
Pre-Scythian (Mezőcsát)	Early Iron Age	4	− 18.1 to − 14.8	− 16.8	1.3	10.4 to 11.0	10.8	0.2
Scythian (Vekerzug)	Early Iron Age	19	− 20.5 to − 15.6	− 17.7	1.1	8.3 to 12.4	9.8	0.9

### Demographic variability

Only adults of known sex were statistically assessed as too few individuals were identified for other demographic groups to be compared (i.e., age groups; Descriptive Information, Supplementary Material S3q; tests of normality, Supplementary Material S3r, S3s, S3t, and S3u). Between periods both females (n = 27) and males (n = 29) exhibit statistical differences for δ^13^C values (Mann–Whitney U: U = 42.5, p = 0.0198, Supplementary Material S3v; independent samples t-test t_27_ = −2.216, p = 0.035, Supplementary Material S3w). There is no statistical difference between Bronze and Iron Age females for δ^15^N (independent samples t-test t_25_ = 1.889, p = 0.070, Supplementary Material S3x), nor for males between periods (independent samples t-test t_27_ = 1.867, p = 0.073, Supplementary Material S3y). However, nineteen individuals remain unsexed. This valuable demographic information, in addition to larger datasets per each demographic group, may alter δ^13^C value results between sexes, and also provide answers to questions concerning the age at which children were incorporated into a social stratification system (e.g., if they reflect more typical adult δ^13^C values^74,75^). No age- or sex-based tests were run by site, culture, or subperiod as there is significant incongruity in sample sizes between and among these categories, which would result in meaningless comparisons.

## Discussion

Two key questions were posed in this research: 1) Is there evidence for nuanced dietary evolution from the Early Bronze Age to Early Iron Age, and 2) if so, what might this imply as concerns communication and trade in the later prehistoric GHP? Broadly speaking, the isotopic data presented here indicate a gradual shift in subsistence strategies from the Early Bronze to Early Iron Age, with evidence for subtle variation between cultures within epochs. In keeping with previous findings, near exclusive consumption of C_3_ plants remains characteristic of the Early Bronze Age^58^. Samples from both the Bronze and Iron Ages largely fall within δ^13^C values typical of C_3_ plant consumers, with a gradual increase in values over time. More specifically, the Late Bronze Age Piliny/Kyjatice samples are the first to exhibit as a whole less negative δ^13^C ratios, indicating a substantial shift from C_3_ plants or aquatic resources to C_4_ plants, concomitant with archaeological evidence for increased millet consumption^51,52^. Early Iron Age pre-Scythian (Mezőcsát) samples continue the Late Bronze Age trend by exhibiting enriched δ^13^C ratios, also in keeping with evidence for heavy reliance on millet at this time^53–55^.

The Bronze and Iron Age samples also exhibit less variable δ^15^N ratios than previous periods^58^. The slightly higher δ^15^N values of the Bronze Age compared to Iron Age samples indicate greater reliance on protein in the former period. Subtle changes between subperiods until the Early Iron Age point to a gradual shift from a more terrestrially omnivorous diet, potentially with a low trophic level aquatic resource influence. Although changes were detected for both males and females among the Bronze and Iron Age periods for δ^15^N ratios, these differences are similar to those seen in the broader pattern between periods (Supplementary Material S3h and S3j). As the pattern of change between the Bronze and Iron Ages is not limited to one sex, this shift in food consumption can be interpreted as occurring among the entire population, as demonstrated by less negative δ^13^C values. These data thus provide evidence for nuanced dietary changes between the Early Bronze to Early Iron Ages. The implications for these findings are addressed below.

### Middle Bronze Age millet consumption

Although there is scattered evidence that broomcorn millet was present in Europe (including present-day Hungary) from the Early Neolithic^51,65,76^, direct radiocarbon dating of millet grains from sites in Central and Eastern Europe disputes its initial economic importance in the human diet, or as a foddering source prior to the Bronze Age^77^. It is hypothesized instead to have been gradually incorporated into subsistence strategies from the Middle to Late Bronze Age^52^. While the AMS ^14^C results of Filipović et al.^78^ challenge this, our smaller dataset continues to indicate its slower incorporation, at least in the GHP. Moreover, though millet has been radiocarbon dated to ~ 1600–1400 BCE at Fajsz 18 (Hungary)^79^, it is otherwise virtually undocumented archaeologically until the Late Bronze Age in the GHP^48,51^. However, our data indicate the presence of millet, or a grain with comparable δ^13^C ratios, may have already begun in the Middle Bronze Age. Specifically, the Füzesabony yielded variable δ^13^C ratios that span both traditional terrestrial C_3_ and C_4_ ranges (Table 3).

Our results are supported by other recent isotopic^80^, radiocarbon^79^, and archaeobotanical^78^ findings. For example, millet grains have been identified in Middle Bronze Age contexts in Moldova, from where it may have spread west up the Danube into the GHP along with other trade items^78,79^. In an isotopic analysis of the contemporaneous Trzciniec culture of Lesser Poland^80^, it was posited that broomcorn millet may have been introduced to the region through cultural interaction with, or migration of, the Otomani-Füzesabony (and/or Tumulus) culture, as suggested by the exchange of culturally diagnostic prestige objects (e.g., beads, pins, amber, maces, ceramics)^47,81–83^. Additionally, broomcorn millet was dated to the Middle Bronze Age at Maszkowice (Poland) where the authors note ceramic and metal artefacts are similar to those recovered in the south Tisza valley within the Otomani-Füzesabony tradition^78^.

A web of long-standing, long-distance trans-Carpathian exchange and communication networks appears to have often followed rivers and tributaries that connected the GHP north via the Vistula, Elbe, and Oder rivers towards the Baltic and North seas, east via the Tisza into Lesser Poland and Ukraine, and south via the Sava, Morava, and Vardar rivers towards the Aegean^38^. Northward dispersal of millet from the GHP may have progressed through such “communication corridors”, together with the exchange of cultural objects and information^47^. Several of our Füzesabony samples derive from the site of Mezőzombor-Községi temető, located near the central Tisza River at the confluence of the GHP and mountains^60^. Another sample, radiocarbon dated to 1740–1440 cal BCE, is from Nagyrozvágy-Papdomb, located on the Bodrog River; the site also has bronze and gold artefacts^60^. Located near rivers, these sites were ideally situated for trade. As noted earlier, increased socio-political and metallurgical complexity in the Carpathian Basin is evidenced by advanced trade networks and communal cemeteries associated with the Otomani-Füzesabony^47^. Furthermore, potential links between the Füzesabony and the introduction of millet, to contemporaneous Middle Bronze Age cultures of Lesser Poland and Ukraine, have been posited^6^ and are corroborated by our data, and archaeological evidence for complex communication and trade networks at this time. However, fully establishing whether millet was adopted or dispersed by the Füzesabony through trade (e.g. as part of a network package from other areas), or by migrants directly introducing this crop to the local GHP population, requires additional genetic and radiocarbon data along with strontium and oxygen isotope approaches. Moreover, data from other Middle Bronze Age GHP cultures (e.g., Tumulus, in prep.), are needed to address whether millet consumption gradually intensified from the Middle Bronze Age in the GHP, as suggested by our results, or if, as posited by Filipović et al.^78^, it became an important crop from the outset.

Lastly, it must be noted that the elevated δ^13^C values of this period may also, at least in part, result from consumption of livestock that had been grazed on C_4_ plants^80^. However, this is a less parsimonious explanation given previous cultures from the same region ought then to also exhibit elevated δ^13^C values if they or their livestock consumed wild C_4_-enriched plants^84^. Moreover, δ^13^C values of fauna (− 21.8 to − 19.4‰ with a mean value of − 20.6‰ ± 0.6‰ (1σ)), we previously obtained from the GHP, are consistent with terrestrial C_3_ environment ranges^57,60^.

### Scythian subsistence economy

In general, the Early Iron Age samples exhibit δ^13^C ratios suggestive of an increase in C_4_ plants, though remain proportionally more C_3_-based. However, the Early Iron Age Scythian (Vekerzug) samples specifically display greater variability, with the reappearance of more negative δ^13^C values, indicating some individuals consumed a mix of C_3_ and C_4_ cereals. They furthermore exhibit a reduction in δ^15^N values in comparison to Early Bronze Age populations and the pre-Scythian (Mezőcsát). This is potentially associated with increased sedentism in some Scythian groups, but greater reliance on pastoralism and thus C_3_ plants, in others^85,86^. Additionally, these samples were consistently different to many other cultures for δ^15^N values; their significantly depleted (mean = 9.8‰) ratios suggest either less animal protein or lower trophic level protein, perhaps due to a focus on agriculture and away from aquatic resources entirely. Broomcorn millet is consistently found in Scythian settlements, and is in general associated with pastoral nomads^87^. For example, at Rákoskeresztúr Újmajor and Ebes Zsong-völgy, barley and broomcorn millet predominate^48,87,88^. Lastly, the Vekerzug also notably differ from the Late Bronze Age Piliny/Kyjatice, the latter of which lived between mountains, a geographic restriction that may have resulted in dietary constraints.

In our dataset only one human sample (HUNG155) shows a δ^13^C signature (− 20.5‰) suggesting heavy reliance on C_3_ plants during the Early Iron Age. This individual also exhibits the most enriched δ^15^N value (12.5‰), significantly higher than other adult individuals, indicating another factor may have resulted in this enrichment. While inflammatory illness (e.g., tuberculosis) could account for this elevation, which in turn may be associated with the depleted δ^13^C values^31^, the identification of disease or infection was not possible due to poor skeletal preservation. Alternatively, this individual may have been engaged in pastoral nomadism, or consumed secondary products from animals grazing C_3_ plants^86,89^. HUNG155 derives from the as yet unpublished Kesznyéten-Szérűskert cemetery, which yielded inhumed remains in a variety of burial positions, indicative perhaps, of both a diverse community and funerary rites^90^. Interestingly, HUNG155 is also a possible instance of corpse mutilation^90^, the significance of which may be further elucidated by the addition of strontium isotope values (in prep.).

Although the Scythians were historically portrayed as a nomadic-pastoralist warrior class, particularly in Central Asia^91,92^, data from Iron Age sites in Eurasia, East-Central Europe, and the GHP point to what appears to be a locally more complex scenario. Despite scarce evidence for Early Iron Age sites in the GHP, which corroborates nomadic pastoralism, recent archaeobotanical^88^, pollen^55^, and now stable isotope findings, challenge the perception of Scythian societies as defined by pastoral nomadism. They instead depict a more complex scenario in which certain groups were nomadic herders, while others engaged in mixed farming or agro-pastoralism, potentially also occupying more settled communities^53,56^. For example, macrofossils of six-row barley and millet were recovered at Rákoskeresztúr-Újmajor in the Alföld^88^, while pollen records dating to the Hungarian Early Iron Age allude to both the intensification of pastoralism and the continued importance of a mixed farming regime, alongside highly developed iron metallurgy and ceramic manufacture^53,55,56^.

Diversification of local economies and adaptation to local environments has been posited based on archaeobotanical data from several Scythian sites in Central Asia^93^, which point to a similarly heterogenous subsistence economy as identified in our dataset. Archaeobotanical evidence for floodplain cereal cultivation of broomcorn millet and hulled barley has been recovered in Ukraine^94^, as has that of wheat, barley, millet, and rye in central Asia^93^ and Russia^95^. Recent isotopic evidence for cereal consumption in Scythian populations has also been reported for sites in Siberia and East Central Europe. For example, the urban Bel’sk (Ukraine) population was found to generally be composed of more sedentary agro-pastoralists who focused on millet cultivation^85,86^. It was also posited that millet and C_3_ cereals may have composed a significant proportion of the diets of two Scythian communities of the Minusinsk and Tuva basins (Siberia), but that consumption of animals foddered on C_3_ plants would isotopically mask their contribution^84^. It remains to be established whether this is associated with increased sedentism. Given genetic evidence^66^ that Scythian groups were comprised of Late Bronze Age herders, farmers, and hunter-gatherers, further stable carbon, nitrogen and strontium isotopes of Early and Middle Iron Age Scythian populations from a dataset derived from diverse cemeteries, will also help to identify potentially heterogenous lifeways within and between Iron Age cultures throughout the Carpathian Basin, and Eurasia at large.

Lastly, it must be noted that manuring affects the δ^15^N values of crops and their consumers^96–98^ with cattle manure altering ratios by + 2 to 8‰, and pig manure by + 15 to 20‰^99^. Given humans, who consume mainly herbivorous animal protein, have an expected δ^15^N range of + 8.5 to 12.5‰, those consuming manured cereals in a mixed plant- and animal-based diet should exhibit a concentrated range between + 6 and 9‰^34,97,100^. Accordingly, our study shows that δ^15^N ratios progressively decrease from the Early Bronze to the Early Iron Age, with a stabilization of values that are likely due to manured crop consumption. The vast majority of our Scythian samples fall within the manured crop consumption range, suggesting a subsistence strategy of some animal protein intake combined with manured crops^34,64,96,97,100^. This indicates more uniform agricultural practices that resulted in more homogenized isotopic values.

## Conclusions

The previously undetected nuanced differences we report here between the isotopic signatures of distinct cultures, and throughout the Early Bronze to Early Iron Ages, demonstrate that dietary evolution remains as complex and nonlinear as the cultural processes, and economic strategies with which it is entangled. The continued amalgamation of research that includes both multi-isotopic and varied archaeological approaches will help shed further light on local and trans-Carpathian subsistence and trade. Lastly, due to the fact that we cover a wide range of cultures throughout a large time frame, some sample sizes are small. Future studies should build upon our results with larger datasets to provide an even higher resolution analysis of the detected trends.

## Material and methods

Stable carbon and nitrogen isotope analyses were conducted on bone samples from 74 human individuals spanning the Early Bronze to the Early Iron Age from the GHP micro-region and the adjacent Northern Mountain Range (Supplementary Material S2). Biological sex of adult individuals was determined based on flexure of the mandibular ramus^101^, and dimorphic traits for the distal humerus^102^, and cranial and postcranial skeleton^103^. Adults were aged according to standard methods for the ilium^104^, pubic symphysis^105^, sternal aspect of ribs^106,107^, and according to obliteration of ectocranial sutures^108^. Subadult age was estimated based on the ossification of apophyseal and epiphyseal joints^109,110^, development of dentition^111^, and diaphyseal long bone measurements^112,113^. Age grouping follows Martin and Saller^114^. When possible, material was assessed for palaeopathological data (Supplementary Material S2).

Collagen extraction was performed at the University College Dublin Conway Institute (Dublin, Ireland) following a modified version of the Longin method^115^, which can be found in detail elsewhere^116–118^. Each sample was weighed to ~ 0.6 mg and placed into a tin capsule. Several samples were processed twice to assure repeatability. All samples were within the acceptable range of two standard deviations of each other^58^. Samples were processed using a Thermo Finnigan DeltaPlus XL mass spectrometer. The accuracy and precision of the measurements, based on repeated measurements of two international laboratory standards USGS40 and USGS41, is ± 0.1‰ (1σ) for δ^13^C and ± 0.1‰ (1σ) for δ^15^N. All carbon stable isotopic results are expressed as a delta (δ) value relative to Vienna Pee Dee Belemnite (VPDB), and all nitrogen stable isotopic results as a delta (δ) value relative to ambient air (AIR).

Samples were assessed for contamination based on carbon and nitrogen content or weight (%). Acceptable %C ranges for modern mammalian bone collagen are between 15.3% and 47%, and for %N between 5.5% and 17.3%; samples falling outside those ranges were deemed inappropriate for analysis^119^. Statistical analyses were performed to assess differences between time periods, demographic groups (i.e., age and sex), and cultures. Statistical analyses were not conducted on certain groups when the number of samples was too few to yield any meaningful analyses (n ≤ 4). Each group was checked for normality using a Shapiro–Wilk test, and equality of variance with Levene’s test, with a p < 0.050 as the statistical significance level. For pairwise comparisons among groups, t-tests (for normally distributed data), and Mann–Whitney U tests (for abnormally distributed data) were employed using p < 0.050 as the statistical significance level. When comparing multiple groups and to determine significant differences between them, one-way ANOVA and Kruskal–Wallis tests were employed for normally and abnormally distributed data, respectively. Post-hoc analyses were performed in cases of significance according to the normality of the data (Tukey’s, Mann–Whitney U, and Bonferroni tests). Statistical data were generated using R (v. 3.6.3^120^) using the ggplot2^121^ package to generate figures.

### Ethics statement

All necessary permits were obtained for the described study, which complied with all relevant regulations and ethical approval (Herman Ottó Múzeum, Miskolc; Dobó István Castle Museum, Eger; Hungarian National Museum, Budapest; Déri Museum, Debrecen; Budapest History Museum—Aquincum Museum and Archaeological Park, Budapest; Damjanich János Museum, Szolnok).

## Supplementary Information

Supplementary Information.

## Data Availability

All data generated or analysed during this study are included in this published article (and its supplementary information files).
