# Supplementary material for: Stable carbon and nitrogen isotopes identify nuanced dietary changes from the Bronze and Iron Ages on the Great Hungarian Plain
Source: Sci Rep. 2022 Oct 10;12:16982. doi: 10.1038/s41598-022-21138-y (PMC9550812; doi:10.1038/s41598-022-21138-y)
Supplement: Supplementary file 1 — Supplementary Information. [file 41598_2022_21138_MOESM1_ESM.pdf]

McCall A, Gamarra C, Duffett Carlson KS, Bernert Z, Cséki A, Csengeri P, Domboróczki L, Endrődi A, Hellebrandt M, Horváth A, Király Á, Kiss K, Koós J, Kovács P, Köhler K, Szolnoki L, Zoffmann ZK, Sirak K, Szeniczey T, Dani J, Hajdu T, & Pinhasi R. Stable carbon and nitrogen isotopes identify nuanced dietary changes from the Bronze and Iron Ages on the Great Hungarian Plain

Supplementary Materials S1 - Archaeological & Burial Data

Legend: IA = Iron Age, BA = Bronze Age, EIA = Early Iron Age, EBA = Early Bronze Age, MBA = Middle Bronze Age, LBA = Late Bronze Age

ID	Period	Sub period	Culture	Site	Location	Grave Number (Feature/ Stratigraphic Number)	Archaeological & Burial Information	References
HUNG81	BA	N/A		Ongaújfalu-Állami gazdaság		Nº2	The Herman Ottó Museum was informed by the state farm of Ongaújfalu that a skeleton had been found during sand mining activity. The site located on N-S sand hill on the edge of the Hernád floodplain. Archaeologist T. Kemenczei travelled to Ongaújfalu on 26 June 1967. The site was a sand mine near the pig farm of the State Farm. There were no traces of other graves. It was probably a solitary grave. It was no longer possible to determine the exact position of the skeleton because of the disturbance, but the two handmade vessels found in the grave can be dated by Kemenczei to the Early Bronze Age (Museum Rep. of HOM 1968; Kemenczei 1968). Later Kemenczei dated this particular burial to the Copper Age (Kemenczei 1969)	Museum Repository of Herman Ottó Museum 269-1968; Kemenczei, T. 1968: Ongafalu /Borsod-Abaúj-Zemplén m., encsi j./ Régészeti Füzetek Ser.I/No.21, Az 1967. év régészeti kutatásai 1968. 12; Kemenczei, T. 1969: A Herman Ottó Múzeum leletmentései és ásatásai 1967-ben. A miskolci Herman Ottó Múzeum Évkönyve VIII., 505-514
HUNG309	BA	EBA	Nyírség	Konyár-Pocsajiműút	southern part of Hajdú-Bihar County (Bihar region, eastern Hungary)	3, Feature 3/Strat. 3	Located on the eastern bank of the Kálló River, preventative excavation of the multi-period site was undertaken in 2011 by L. Szolnoki (Déri Museum, Debrecen). HUNG309 was found in a slightly contracted position oriented S-N on their right side. The skull has moved from its original position, which suggests that the grave was disturbed. The deceased was a 15-17 years old gracile female based on the primary anthropological examination (Zoffmann, 2013). The incisors and canines show signs of hypoplasia, and one tooth (left side PM2) was surgically removed during her lifetime. The grave pit is an irregular rectangle, 70-77 cm deep. The body was surrounded by two groups of ceramics: three vessels (a broom decorated deep bowl, a jar and a broom decorated little bowl), located behind her head and two others (a mug and a broom decorated pot) around her heels. Skeletal remains of an adult sheep, which may have been a meat offering, were found behind the spine. Based on ceramic typology the grave is identified as a Nyírség inhumation burial dating to the Early Bronze Age.AMS radiocarbon dating of this grave was made in the Debrecen HEKAL Radiocarbon Laboratory (DeA-2799), which gave 3642 ±33 BP conventional radiocarbon age. After the calibration we get 2136-1901 calBC 2 sigma range.	The Archaeological Repository of the Déri Museum, Debrecen; K. Zoffmann, Zs. 2013: Bronzkori embertani leletek a Kárpát-medence keleti térségeiből. (Adatközlés). / Anthropological artefacts from the Bronze Age from the eastern region of the Carpathian basin (Data statement). A Debreceni Déri Múzeum Évkönyve 2013, 29-39
HUNG494	BA	EBA	Proto-Nagyrév	Szigetszentmiklós-Üdülősor	Csepal Island, 3km from Budapest	458	Rescue excavations of 2,500 m2 area were conducted between 1988-1989 by motorway M0; later 2008 salvage excavations were undertaken during M0 motorway expansion. Occupying a wide elevated area in the Danube floodplain, ~100m from the Soroksár branch of the Danube, the site is associated with, for example, the MCA, LCA, Bell Beaker, and Medieval Árpádian cultures. Excavation unearthed houses, refuse pits and graves. The dated EBA II/b assemblage corresponds to the early Nagyrév culture. At this point there was interaction, including assimilation between the early Nagyrév and Bell Beaker cultures, which began circa the EBA II/a and ceased at the end of the early Nagyrév period. In total 67 graves were unearthed at the site, four of which were in an unusual position, and only one of which (of the four) contained an associated artefact: an early Nagyrév cup placed mouth downwards in Feature 12 next to a body in face-down position. The burials in unusual positions form a cluster at the periphery of the cemetery, and are separate from those recovered from the Beaker-Csepel burial area. This, in conjunction with the mouth-downwards facing vessel in Feature 12, have led to the suggestion that the early Nagyrév people may have been given enemy status by the Bell Beaker population, which controlled stategic land around the Danube.	Endrődi, A. Recent Data of the Settlement History and Contact System of Bell Beaker-Csepel Group. Ősrégészeti tanulmányok/Prehistoric Studies I. In Moments in Time" Papers presented to Pál Raczky on His 60 th Birthday (eds. Anders, A. & Kulcsár, G.) 693–707 (2013); K. Zoffmann, Z. A Harang alakú edényének népének embertani leletei Csepel szigetről - Anthropological finds of the Bell Beaker Culture from Csepel Island. Anthropol. Kozl. 47, 11–15 (2006); Olade et al., 2018
HUNG495	BA	EBA	Proto-Nagyrév	Szigetszentmiklós-Üdülősor		12/S25		

Legend: IA = Iron Age, BA = Bronze Age, EIA = Early Iron Age, EBA = Early Bronze Age, MBA = Middle Bronze Age, LBA = Late Bronze Age

ID	Period	Sub period	Culture	Site	Location	Grave Number (Feature/ Stratigraphic Number)	Archaeological & Burial Information	References
HUNG496	BA	EBA	Proto-Nagyrév	Szigetszentmiklós-Üdülősor		244/S26		
HUNG497	BA	EBA	Proto-Nagyrév	Szigetszentmiklós-Üdülősor		119/S22		
HUNG498	BA	EBA	Bell Beaker	Szigetszentmiklós-Üdülősor		13		
HUNG499	BA	EBA	Bell Beaker	Szigetszentmiklós-Üdülősor		1 (10.05.1988)		
HUNG360	BA	EBA	Hatvan	Apc-Berekalja I	Near the Zagyva Valley (border of Pest and Heves Counties)	928/13	Located at the easter periphery of western LBK lands near the limit of the eastern Alföld Linear Pottery (ALP) culture. Structures including LBK houses, graves and pits were recovered as were LCA, EBA, MBA and Roman features. Graves were unearthed dating to numerous periods, including the BA. Grave 928/13 (HUNG 360), in which a contracted skelton on their left side was recovered in a deep pit, dates to the Late Hatvan period.	Gamarra et al., 2018
HUNG370	BA	EBA/MBA	Hatvan or Füzesabony	Kompolt-Kígyósér	between Kál and Nagyút (Heves County, eastern Hungary)	07-Mar	1994 excavations covering 24,000 m2 resulted in the recovery of numerous artefacts dating from the Neolithic to Middle Ages. Grave 7/3 (HUNG370) was found in the Neolithic ALP grave group; however, despite the grave containing a ceramic pot typical of ALP material, two radiocarbon dates identify the skeleton as belong to the BA. The individual comprises a complete skeleton found in a crouched position on its left side.	Gamarra et al., 2018
HUNG933	BA	MBA	Hatvan or Füzesabony	Vatta-Dobogó	south of Vatta Village (Borsod-Abaúj-Zemplén County, NE Hungary).	S109	Excavated during a water management and stream bed relocation project. Located at the confluence of the GHP and mountains, in the Bükkábrány lignite mine region, next to Vatta-Testhalom, a BA tell. In the outer region of the settlement a rampart associated with the EBA Hatvan and MBA Füzesabony cultures. Neolithic, EBA, and MBA burials were also recovered as were artefacts dating to the LBA Piliny and Roman Sarmatian cultures.	Hernando et al., 2021
HUNG934	BA	MBA	Hatvan or Füzesabony	Vatta-Dobogó		S257/II		
HUNG935	BA	MBA	Hatvan or Füzesabony	Vatta-Dobogó		S169		
HUNG936	BA	MBA	Hatvan or Füzesabony	Vatta-Dobogó		S279		
HUNG938	BA	MBA	Hatvan or Füzesabony	Vatta-Dobogó		S166		
HUNG721	BA	MBA	Otomani/Ottomány	Ófehértó-Almezői dűlő (site M3 210)	SW boundary of Ófehértó (Szabolcs-Szatmár-Bereg County, NE Hungary)	100/S186	Located approximately in the middle of a NNW-SSE sand dune in the Nyírség geographical region, bordered on the west by a marshy meadow called "Bika-rét". The multi-period NNW oriented site is extensive. The grave pit is an irregular rectangle cut into the sandy subsoil. The skeleton lies in a contracted position on its left side (the right side of the skeletal system was almost completely destroyed during humus removal, except for the leg bones). The skull is also tilted to the left side, but is displaced from its original position and is located near the southeast corner, and it was also damaged by the machine. Some of the vertebrae and ribs are missing. The severely incomplete arm bones were originally bent at the base of the left humerus. The leg bones are drawn up to the pelvis and were crossed at about knee height. The fibulae and the bones of the right foot are missing and there was found a bovine horn fragment next to the left foot. Several ceramic fragments and animal bones were recovered from the fill of the gravepit. Although there is no radiocarbon dating, based on the ceramic fragments from the gravepit the burial can be dated to the Middle Bronze Age (Otomani culture).	Cséki, A. 2010 Ófehértó, Almezői-dűlő 18-19. Tájékoztató a K.Ö.SZ. 2009. évi tevékenységéről, Budapest (2010) 29; Cséki, A. 2012 Ófehértó, Almezői-dűlő (Szabolcs-Szatmár-Bereg megye). Régészeti kutatások Magyarországon 2010. Budapest (2012) 313-314; Cséki, A. - Füzesi, A 2012 Ófehértó határa (Szabolcs-Szatmár-Bereg megye). Almezői-dűlő. Régészeti kutatások Magyarországon 2011. Budapest (2018) 129-131; Cséki, A. 2012 Ófehértó, Almezői-dűlő. Évkönyv és jelentés a K.Ö.SZ. 2009. évi feltárásairól. Budapest (2012) 11-12; Cséki, A. 2013 Ófehértó, Almezői-dűlő M3 253. Tájékoztató a MNM NÖK 2010-2011. évi tevékenységéről, Budapest (2013) 38
HUNG127	BA	MBA	Füzesabony	Mezőzombor-Községi temető	41 km east of Miskolc (Borsod-Abaúj-Zemplén County, NE Hungary).	4/2000 (2006.70.8)	A rescue excvavation was conducted after grave-digging in a local cemetery; eight MBA skeletons in crouched positions were found and dated via artefacts to the Classical Füzesabony period. Located in the central Tisza area at the confluence of the GHP and mountains, findings dating to multiple periods (e.g., Neolithic, CA, EBA (Nyírség) and LBA (Kyjatice)) were found.	Hernando et al., 2021

Legend: IA = Iron Age, BA = Bronze Age, EIA = Early Iron Age, EBA = Early Bronze Age, MBA = Middle Bronze Age, LBA = Late Bronze Age								
ID	Period	Sub period	Culture	Site	Location	Grave Number (Feature/ Stratigraphic Number)	Archaeological & Burial Information	References
HUNG128	BA	MBA	Füzesabony	Mezőzombor-Községi temető		3/2000 (2006.70.7)		
HUNG129	BA	MBA	Füzesabony	Mezőzombor-Községi temető		2/2000 (2006.70.6)		
HUNG130	BA	MBA	Füzesabony	Mezőzombor-Községi temető		16/2000 (2006.70.5)		
HUNG131	BA	MBA	Füzesabony	Mezőzombor-Községi temető		41/2001		
HUNG132	BA	MBA	Füzesabony	Mezőzombor-Községi temető		6/2000 (2006.70.10)		
HUNG133	BA	MBA	Füzesabony	Mezőzombor-Községi temető		31/2001		
HUNG134	BA	MBA	Füzesabony	Mezőzombor-Községi temető		10/2001 (2006.71.1)		
HUNG135	BA	MBA	Füzesabony	Mezőzombor-Községi temető		56/2001		
HUNG136	BA	MBA	Füzesabony	Mezőzombor-Községi temető		57/2001		
HUNG147	BA	MBA	Füzesabony	Mezőkeresztes-Csincse-tanya	Csincse Village, 25 km from Miskolc (Southern Borsod-Abaúj-Zemplén County, NE Hungary)	1	The site is located in the Füzesabony core area. A skeleton in a crouched position, with three artefacts, including a bowl and other vessels was found 1 m deep inside a hydrologic observation well in 1968. The associated artefacts date the skeleton to the classical Füzesabony period.	Wolf, M. & Simonyi, E. Mezőkeresztes-Cethalom: Kora népvándorláskori temető az V-VI. századból / Gepidic cemetery at Mezőkeresztes-Cethalom. in Path into the Past (eds. Raczky, P., Kovács, T. & Anders, A.) 128–132 (1997); Hernando et al., 2021.
HUNG163	BA	MBA	Füzesabony	Nagyrozvágy-Papdomb	NortheasternBorsod-Abaúj-Zemplén County, by Slovakian border	296	Largescale excavations ran from 2005 to 2007 during construction of an emergency reservoir in the floodplain area of the Bodrog River. A settlement dating to the Late Bodrogszerdahelyi (Streda nad Bodrogom) phase of the Füzesabony culture (MBA, radiocarbon dated to 1740-1440 cal BCE). Finds included bronze and gold artefacts, household objects, and features like houses and fountains. Human remains were found scattered in burials as were three individuals.	Hernando et al., 2021
HUNG142	BA	LBA	Piliny	Mezőkeresztes	Southern Borsod-Abaúj-Zemplén County, NE Hungary	Szorv		
HUNG380	BA	LBA	Piliny/Kyjatice	Ludas-Varjú-dűlő	north of Ludas (Heves County)	2181	Located in the foothills of the Mátra Mountain. Excavations from 1998-2002 unearthed an 18 ha, rich LBA settlement, radiocarbon dated to 1540-1000 cal BCE. The site includes a round monumental ditch and palisade on the eastern side that may have acted as a ceremonial centre. An urn cemetery was found at the northern end of the site. Although no houses were recovered from the inner settlement, numerous pits were preserved, over 20 of which contained human burials. Both radiocabron dating of human skeletal material and ceramic typology suggest the burials are Kyjatice.	Domboróczki L. Régészeti-kutatások Ludas, Varjú-dűlőn 1998 és 2002 között (Előzetes jelentés) — Archaeological investigations at Ludas, Varjú-dűlő between 1998 and 2002 (Preliminary Report). Régészeti Kut Magyarországon 2002/Archaeological Investigations Hungary 2002. 2004; 5–23; Domboróczki L. Recherches archéologiques à Ludas-Varjú-dűlő. In: Szabó M, editor. La nécropole celtique à Ludas-Varjú-dűlő. L'Harmattan; 2012. pp. 155–169; Gamarra et al., 2018
HUNG387	BA	LBA	Piliny/Kyjatice	Ludas-Varjú-dűlő	north of Ludas (Heves County)	1058/9	HUNG 387, a highly fragmented skeleton, was excavated from a pit (1058) just 16 cm deep, a secondary burial; the burial had associated cattle remains. Human remains belonging to other individuals were also found out of context in ashy or charred layers in Pit 1058, suggesting cremation prior to burial.	Gamarra et al., 2018
HUNG392	BA	LBA	Piliny/Kyjatice	Ludas-Varjú-dűlő	north of Ludas (Heves County)	1433	HUNG 392 was excavated from Pit 1433.	Gamarra et al., 2018
HUNG397	BA	LBA	Piliny/Kyjatice	Ludas-Varjú-dűlő	north of Ludas (Heves County)	1936/1	HUNG 397 was excavated from Pit 1936 with a second individual.	Gamarra et al., 2018
HUNG406	BA	LBA	Piliny/Kyjatice	Ludas-Varjú-dűlő	north of Ludas (Heves County)	1851	HUNG 406, a complete skeleton in a crouched position on its left side, was excavated from the lowest part of Pit 1851 and was richly provisioned with grave goods, including vessels found on top of the body, which was close to the pit walls.	Gamarra et al., 2018

Legend: IA = Iron Age, BA = Bronze Age, EIA = Early Iron Age, EBA = Early Bronze Age, MBA = Middle Bronze Age, LBA = Late Bronze Age								
**ID**	**Period**	**Sub period**	**Culture**	**Site**	**Location**	**Grave Number (Feature/ Stratigraphic Number)**	**Archaeological & Burial Information**	**References**
HUNG410	BA	LBA	Piliny/Kyjatice	Ludas-Varjú-dűlő	north of Ludas (Heves County)	1916	HUNG 410, found in a crouched position on their left side, was excavated from Pit 1916.	Gamarra et al., 2018
HUNG415	BA	LBA	Piliny/Kyjatice	Ludas-Varjú-dűlő	north of Ludas (Heves County)	1935	HUNG 415 was excavated from the lower layer of Pit 1935 with a second individual; the skeletons were fragmented and not in any anatomical position.	Gamarra et al., 2018
HUNG429	BA	LBA	Piliny/Kyjatice	Ludas-Varjú-dűlő	north of Ludas (Heves County)	2181	HUNG 429, found in a crouched position on their back with the legs slightly to the right, was excavated from Pit 2181.	Gamarra et al., 2018
HUNG381	BA	LBA	Piliny/Kyjatice	Ludas-Varjú-dűlő	north of Ludas (Heves County)	2161.2	HUNG 381, radiocarbon dated to 3,130 +- 70 uncal BP, was unearthed from Pit 2161and also included an adult female.	Gamarra et al., 2018
HUNG382	BA	LBA	Piliny/Kyjatice	Ludas-Varjú-dűlő	north of Ludas (Heves County)	1033/8	HUNG 382, a skeleton in a crouched position on their right side, was excavated from Pit 1033.	Gamarra et al., 2018
HUNG407	BA	LBA	Piliny/Kyjatice	Ludas-Varjú-dűlő	north of Ludas (Heves County)	1681/2	HUNG 407 was unearthed from Pit 1681 and also included the remains of a 6-7 year old child (HUNG 412).	Gamarra et al., 2018
HUNG412	BA	LBA	Piliny/Kyjatice	Ludas-Varjú-dűlő	north of Ludas (Heves County)	1681/1	HUNG 412 was unearthed from Pit 1681 and also included the remains of a woman (HUNG 407).	Gamarra et al., 2018
HUNG413	BA	LBA	Piliny/Kyjatice	Ludas-Varjú-dűlő	north of Ludas (Heves County)	1090	HUNG 413, which included the burnt and fragmented remains of a jaw, teeth and long bones, was unearthed from the 20 cm deep Pit 1090.	Gamarra et al., 2018
HUNG137	BA	LBA	pre-Gáva Period	Felsődobsza- site 2	Hernád Valley (Borsod-Abaúj-Zemplén county, Northeastern Hungary)	S62	Excavation began in 2012 by Miklós Makoldi; the site covers >2 hectares and 4 chronological periods: Middle Neolithic, Germanic, Medieval, and Late Bronze Age (~1400/1300-900/850 BCE: extensive settlment occupying the majority of the site, during which time >150 features were built on the site.	Makoldi, M. Felsődobsza 2. lelőhely. Régészeti Kutatások Magyarországon in Archaeological Investigations in Hungary. Eds. Kvassay, J. & Kreiter, A., Hungarian National Museum, 2012
HUNG144	BA	LBA	pre-Gáva Period	Oszlár-Nyárfaszög (M3- site 32)	SE border of Oszlár Village (Southern Borsod-Abaúj-Zemplén county, Northeastern Hungary)	1010	Largescale excavations ran from 1996-1997 as a response to the M3 motorway construction, it was one of the largest LBA excavations in recent times. Situated on the northern border of the GHP, bordered by the Hejő and Tisza rivers, the settlement was inhabited by numerous cultures, including the Carpathian Tumulus culture, Piliny, and pre-Gáva (RB BD - HA1) cultures. Amongst other human remains, EBA (Makó) cremations and LBA burial pits were unearthed.	Hernando et al., 2021
HUNG967	BA	LBA	pre-Gáva Period	Pácin-Alsókenderszer	Pácin Village (Borsod-Abaúj-Zemplén county, Northeastern Hungary)	S64A	Preventative excavation of the site was undertaken in light of floodplain reservoir construction. 2005-2006 excavation by Emese Lovász unearthed an LBA settlement with associated human skeletons, and included two features, and urn graves. Human remains in Feature Nr. 64 were AMS radiocarbon dated to 1192-977 cal BCE (calibrated by using Calib 7.0.4, lab code: DeA-11753).	Hernando et al., 2021
HUNG968	BA	LBA	pre-Gáva Period	Pácin-Alsókenderszer		S64B		
HUNG969	BA	LBA	pre-Gáva Period	Pácin-Alsókenderszer		S100		
HUNG177	BA	LBA	pre-Gáva Period	Mezőkeresztes-Cet halom (site M3-10)		154		
HUNG175	IA	EIA	pre-Gáva Period R BD - Ha A1	Mezőkeresztes-Cet halom (site M3-10)	Southern Borsod-Abaúj-Zemplén County, NE Hungary	22	Rescue excavation of 6000 m2 of the site, which sits at an elevavation over the Lator Stream (~15km north of Lake Tisza), began in 1993 under Mária Wolf prior to M3 motorway construction. Excavations unearthed 26 graves dating to numerous periods (e.g., Middle Neolithic, Late Copper Age, Late Bronze Age, Celtic), and 264 settlement features. In 1995 excavations in the southeast part of town unearthed an LBA Gava culture settlement. A bell-shaped pit (Feature nr. 154) included, along with a feer antler, the disturbed graves of a child and dog. Remains of the child were AMS radiocarbon dated to 1379 and 1130 cal BCE (calibrated by using Calib 7.0.4, lab code: DeA-11654).	Wolf, M. and Simonyi, E. 1995. Előzetes jelentés az M-3-as autópálya 10. lelőhelyének feltárásáról. Autobahn M3 10. Fundstelle. Somogyi Múzeumok Közleményei 11: 5–32; Hernando et al., 2021

Legend: IA = Iron Age, BA = Bronze Age, EIA = Early Iron Age, EBA = Early Bronze Age, MBA = Middle Bronze Age, LBA = Late Bronze Age								
ID	Period	Sub period	Culture	Site	Location	Grave Number (Feature/ Stratigraphic Number)	Archaeological & Burial Information	References
HUNG863	BA	LBA	Gáva	Köröm-Kápolnadomb	Near the floodplain of the Sajó Stream (Borsod-Abaúj, Zemplén County, NE Hungary)	S67	!969 rescue excavations by Tibor Kemenczei unearthed an LBA Gáva site with fifteen features, dating to 1200/1100-900-850 BCE. The 1996 rescue exavation unearthed 85 features, including 20 pits, 11 clay extracting pits, and 5 houses dating to the Gáva period. The 2014 rescue excavation by Zsolt Gallina also unearthed 39 LBA pits. Along with burials dating to later periods, two further skeletons found in atypical positions in Feature no. SNR 020 (a beehive shaped pit), radiocarbon dated by associated fauna to 1191-944 cal BCE (AMS C14 date, calibrated by using Calib 7.0.4, lab code: DeA-11635).	Hellebrandt, M. B. A settlement of the Gáva Culture on Köröm-Kápolna-domb in Yearbook of the Herman Ottó Museum ,11-124 (2016); Kemenczei, T. Die Spätbronzezeit Nordostungarns: Archaeologia Hungarica. (Series Nova L I, Akadémiai Kiado, 1984)
HUNG178	BA	LBA	N/A	Mezőkeresztes-Cet halom (site M3-10)		19		
HUNG401	IA	EIA	Pre-Scythian (Mezőcsát)	Ludas-Varjú-dűlő	north of Ludas (Heves County)	2633	Grave 2633 (HUNG 401) was found oriented W-E on their back, buried in a 35 cm layer of humus.	Gamarra et al., 2018
HUNG417	IA	EIA	Pre-Scythian (Mezőcsát)	Ludas-Varjú-dűlő	north of Ludas (Heves County)	2630	Grave 2630 (HUNG 417) was found oriented SW-NE in an extended position on their back; the grave included a bronze pin and ceramic vessel.	Gamarra et al., 2018
HUNG418	IA	EIA	Pre-Scythian (Mezőcsát)	Ludas-Varjú-dűlő	north of Ludas (Heves County)	2638	A pre-Scythian cemetery was unearthed at the southern edge of the settlement with two groups of skeletons. Many graves contained ceramic and iron artefacts that date the cemetery to the EIA Mezőcsát culture.	Gamarra et al., 2018
HUNG425	IA	EIA	Pre-Scythian (Mezőcsát)	Ludas-Varjú-dűlő		4		
HUNG82	IA	EIA	Scythian Age (Vekerzug)	Kesznyéten-Szérűskert	(Borsod-Abaúj-Zemplén County, NE Hungary)	3	Eighty-nine graves were excavated in 1984-1985 and 1987-1989, thirty-six of which are published. Two main rites, inhumations and cremations were observed. The cemetery is dated to the 5th century BCE. Scythian (Vekerzug culture) elements dominante the archaeological assemblage, but many finds can be connected to the Celts.	(B. Hellebrandt, Magdolna: Szkítakori temető Kesznyéten-Szérűskerten. 1984-85. évi ásatás eredménye - Skythenzeitliches Gräberfeld in Kesznyéten-Szérűskert (Grabungsergebnisse der Jahre 1984-85). Herman Ottó Múzeum Évkönyve 25-26, 1988, 107-126
HUNG145	IA	EIA	Scythian Age (Vekerzug)	Kesznyéten-Szérűskert		38		
HUNG146	IA	EIA	Scythian Age (Vekerzug)	Kesznyéten-Szérűskert		39		
HUNG148	IA	EIA	Scythian Age (Vekerzug)	Kesznyéten-Szérűskert		3		
HUNG149	IA	EIA	Scythian Age (Vekerzug)	Kesznyéten-Szérűskert		53		
HUNG150	IA	EIA	Scythian Age (Vekerzug)	Kesznyéten-Szérűskert		61		
HUNG151	IA	EIA	Scythian Age (Vekerzug)	Kesznyéten-Szérűskert		74		
HUNG152	IA	EIA	Scythian Age (Vekerzug)	Kesznyéten-Szérűskert		78		
HUNG154	IA	EIA	Scythian Age (Vekerzug)	Kesznyéten-Szérűskert		83		
HUNG155	IA	EIA	Scythian Age (Vekerzug)	Kesznyéten-Szérűskert	(Borsod-Abaúj-Zemplén County, NE Hungary)	24	Grave 24 (HUNG155) was found in an extended position in an inhumation burial without grave goods. According to M. Hellebrandt, this is a possible instance of corpse mutilation (Museum inventar number of the deceased of Grave 24: 2002.2.13.	B. Hellebrandt, Magdolna: Szkítakori temető Kesznyéten-Szérűskerten. 1984-85. évi ásatás eredménye - Skythenzeitliches Gräberfeld in Kesznyéten-Szérűskert (Grabungsergebnisse der Jahre 1984-85). Herman Ottó Múzeum Évkönyve 25-26, 1988, 107-126
HUNG156	IA	EIA	Scythian Age (Vekerzug)	Kesznyéten-Szérűskert		9		
HUNG158	IA	EIA	Scythian Age (Vekerzug)	Kesznyéten-Szérűskert		15		
HUNG159	IA	EIA	Scythian Age (Vekerzug)	Kesznyéten-Szérűskert		29		
HUNG160	IA	EIA	Scythian Age (Vekerzug)	Kesznyéten-Szérűskert		28		

Legend: IA = Iron Age, BA = Bronze Age, EIA = Early Iron Age, EBA = Early Bronze Age, MBA = Middle Bronze Age, LBA = Late Bronze Age								
**ID**	**Period**	**Sub period**	**Culture**	**Site**	**Location**	**Grave Number (Feature/ Stratigraphic Number)**	**Archaeological & Burial Information**	**References**
HUNG161	IA	EIA	Scythian Age (Vekerzug)	Kesznyéten-Szérűskert		1		
HUNG162	IA	EIA	Scythian Age (Vekerzug)	Kesznyéten-Szérűskert		2		
HUNG957	IA	EIA	Scythian Age (Vekerzug)	Szikszó-Hell Ring, site 1	(NE Hungary)	S156E	Located in the North Hungarian mountains, outside the core area of the Vekerzug culture. The site remains unpublished. However, the excavated area of the settlement may have been part of a working area, probably connected to pottery making. During the excavation mostly houses and wells were unearthed. Human skeletons were found in archaeological features. This part of the settlement was probably used during the 5th c. BCE. It was excavated in 2016.	Király Ágnes: Beszámoló a szikszói szkíta kori fazekastelep (- részlet) feltárásáról - Magyar Régészet. 2017 tavasz, 1-7
HUNG958	IA	EIA	Scythian Age (Vekerzug)	Szikszó-Hell Ring, site 1		S217		
HUNG960	IA	EIA	Scythian Age (Vekerzug)	Szikszó-Hell Ring, site 1		S461		

Supplementary Materials S2 - Isotopic & Sample Data

Legend: F = female, M = male, N/A = not assessable; IA = Iron Age, BA = Bronze Age, EIA = Early Iron Age, EBA = Early Bronze Age, MBA = Middle Bronze Age, LBA = Late Bronze Age;
LEH = linear enamel hypoplasia, PO = porotic hyperostosis, CO = cribra orbitalia, Y = Yes, N = No

ID	$\delta^{13}C$ ‰ (VPDB)	$\delta^{15}N$ ‰ (AIR)	% Colla-gen	% C	% N	C:N	Sample Type	Age (group)	Age (circa)	Sex	LEH (Y/N)	PO (Y/N)	CO (Y/N)	Skeletal Element	Source
HUNG81	-17.3	9.6	12.40	44.0	15.7	2.9	Human	Mature	40 to 59	F	N/A			long bone	This study
HUNG309	-20.2	9.3	17.58	42.9	15.4	2.9	Human	N/A						metatarsal	This study
HUNG494	-19.8	11.3	7.75	43.9	15.2	2.9	Human	Infant II	11 to 12	N/A				cranium	This study
HUNG495	-19.8	12.9	4.19	39.8	13.8	3.1	Human	Adult	20 to 25	M	N/A			cranium	This study
HUNG496	-19.7	10.7	4.09	44.7	15.7	3.0	Human	Infant II	10 to 12	N/A				cranium	This study
HUNG497	-19.8	12.0	4.85	43.9	15.6	2.9	Human	Infant II	12 to 14	N/A				cranium	This study
HUNG498	-20.3	9.4	1.08	42.3	14.7	3.0	Human	Mature-Senior	50 to 69	M	N/A			cranium	This study
HUNG499	-20.2	9.8	2.65	41.1	14.4	2.9	Human	N/A		F	N/A			cranium	This study
HUNG360	-20.0	10.7	2.35	42.6	15.3	2.9	Human	Mature	40 to 59	M	N/A			long bone	Gamarra *et al.*, 2018
HUNG370	-20.1	10.3	4.18	44.5	16.1	2.9	Human	Adult	31 to 40	F	N/A			rib	Gamarra *et al.*, 2018
HUNG933	-17.2	9.5	3.64	41.5	14.5	3.0	Human	Infant II	8 to 13	F	N	N	N	cranium	This study
HUNG934	-17.5	10.3	6.06	31.7	11.3	2.9	Human	Adult	20 to 39	N/A	Y	N/A	N	femur	This study
HUNG935	-17.8	9.5	23.50	44.7	15.9	3.0	Human	Adult-Mature	20 to 59	N/A		N	N	long bone	This study
HUNG936	-21.0	11.3	4.89	20.6	6.8	3.4	Human	Mature	40 to 59	M	N/A			long bone	This study
HUNG938	-18.1	9.4	11.94	44.5	15.8	3.0	Human	Adult	20 to 39	F	N	N	N	rib	This study
HUNG721	-20.2	9.2	13.49	44.9	16.1	2.9	Human	Juvenile	15 to 17	N/A				rib	This study

Legend: F = female, M = male, N/A = not assessable; IA = Iron Age, BA = Bronze Age, EIA = Early Iron Age, EBA = Early Bronze Age, MBA = Middle Bronze Age, LBA = Late Bronze Age;
LEH = linear enamel hypoplasia, PO = porotic hyperostosis, CO = cribra orbitalia, Y = Yes, N = No

ID	$\delta^{13}C$ ‰ (VPDB)	$\delta^{15}N$ ‰ (AIR)	% Colla-gen	% C	% N	C:N	Sample Type	Age (group)	Age (circa)	Sex	LEH (Y/N)	PO (Y/N)	CO (Y/N)	Skeletal Element	Source
HUNG127	-20.2	11.5	10.77	43.5	15.4	2.9	Human	Adult	20 to 39	F	Y	N	N	metatarsal	This study
HUNG128	-19.9	10.7	3.92	42.5	15.2	2.9	Human	Infant II	8 to 13	N/A	Y	N	N/A	metatarsal	This study
HUNG129	-19.5	11.3	17.98	43.2	15.3	2.9	Human	Adult-Mature	20 to 59	M	N	N	N/A	metacarpal	This study
HUNG130	-20.2	11.2	8.11	43.0	15.6	2.9	Human	Adult	20 to 39	F	Y	N	Y	metatarsal	This study
HUNG131	-20.6	12.3	10.14	42.4	15.1	2.9	Human	Adult-Mature	20 to 59	M	N/A			metacarpal	This study
HUNG132	-20.3	9.0	10.28	43.2	15.4	2.9	Human	Infant I-II	5 to 7	F	N	N	N	metatarsal	This study
HUNG133	-20.2	11.3	17.85	42.2	15.0	2.9	Human	Adult	20 to 39	M	N/A			metatarsal	This study
HUNG134	-20.2	11.7	4.50	42.4	15.4	2.9	Human	Infant I-II	5 to 10	M	Y	N	N/A	metatarsal	This study
HUNG135	-20.0	11.0	37.87	42.5	15.3	2.9	Human	Adult-Mature	20 to 59	M	N/A	N	N	metatarsal	This study
HUNG136	-20.0	11.3	10.05	43.6	15.6	2.9	Human	Adult-Mature	33 to 46	F	Y	N	N/A	metatarsal	This study
HUNG147	-16.8	10.6	4.85	43.0	15.4	2.9	Human	Infant II	12 to 14	N/A	N	N	Y	metacarpal	This study
HUNG163	-21.2	11.1	8.00	42.9	14.1	3.1	Human	Adult-Mature	35 to 45	M	N/A	N	N/A	metacarpal	This study
HUNG142	-19.5	10.6	5.31	43.9	15.5	2.9	Human	N/A			N/A			metatarsal	This study

Legend: F = female, M = male, N/A = not assessable; IA = Iron Age, BA = Bronze Age, EIA = Early Iron Age, EBA = Early Bronze Age, MBA = Middle Bronze Age, LBA = Late Bronze Age; LEH = linear enamel hypoplasia, PO = porotic hyperostosis, CO = cribra orbitalia, Y = Yes, N = No															
ID	$\delta^{13}C$ ‰ (VPDB)	$\delta^{15}N$ ‰ (AIR)	% Colla-gen	% C	% N	C:N	Sample Type	Age (group)	Age (circa)	Sex	LEH (Y/N)	PO (Y/N)	CO (Y/N)	Skeletal Element	Source
HUNG380	-19.8	10.8	10.37	44.0	15.6	2.9	Human	N/A						rib	This study
HUNG387	-18.7	11.2	2.77	43.0	15.3	2.9	Human	Adult	20 to 39	M	N/A			rib	Gamarra *et al.*, 2018
HUNG392	-17.1	10.2	5.52	43.3	15.2	2.9	Human	Adult	20 to 39	M	N/A			cranium	Gamarra *et al.*, 2018
HUNG397	-18.1	11.1	9.49	42.9	14.9	3.1	Human	Mature	40 to 59	F	N/A			cranium	Gamarra *et al.*, 2018
HUNG406	-19.0	10.8	5.26	43.2	15.7	2.9	Human	Infant I-II	1 to 13	N/A	N/A			rib	Gamarra *et al.*, 2018
HUNG410	-17.4	10.3	3.43	42.2	14.8	3.0	Human	Adult	20 to 39	F	N/A			long bone	Gamarra *et al.*, 2018
HUNG415	-17.4	10.5	12.95	42.3	15.1	2.9	Human	Juvenile-Adult	15 to 39	M	N/A			metacarpal	Gamarra *et al.*, 2018
HUNG429	-18.1	11.5	4.27	43.5	15.3	2.9	Human	Adult	35 to 39	M	N/A			rib	Gamarra *et al.*, 2018
HUNG381	-16.2	12.8	1.62	35.0	12.5	2.9	Human	Infant I	2 to 3	M	N/A			cranium	Gamarra *et al.*, 2018
HUNG382	-17.7	11.2	6.81	42.7	15.2	2.9	Human	Adult	20 to 29	M	N/A			rib	Gamarra *et al.*, 2018
HUNG407	-17.3	11.6	5.20	43.2	15.7	2.9	Human	Juvenile-Adult	15 to 39	F	N/A			long bone	Gamarra *et al.*, 2018
HUNG412	-18.6	11.0	4.06	42.7	14.6	3.1	Human	Infant I	1 to 6	N/A	N/A			rib	Gamarra *et al.*, 2018
HUNG413	-17.6	11.1	4.15	41.4	14.8	3.0	Human	Adult	20 to 39	M	N/A			long bone	Gamarra *et al.*, 2018
HUNG137	-15.1	10.8	19.73	42.8	15.4	2.9	Human	Adult-Mature	20 to 59	M	Y	N	N	metatarsal	This study
HUNG144	-16.8	10.9	4.53	43.9	15.6	2.9	Human	Adult	20 to 39	M	N/A			metacarpal	This study

Legend: F = female, M = male, N/A = not assessable; IA = Iron Age, BA = Bronze Age, EIA = Early Iron Age, EBA = Early Bronze Age, MBA = Middle Bronze Age, LBA = Late Bronze Age; LEH = linear enamel hypoplasia, PO = porotic hyperostosis, CO = cribra orbitalia, Y = Yes, N = No															
**ID**	**δ^{13}C ‰ (VPDB)**	**δ^{15}N ‰ (AIR)**	**% Colla-gen**	**% C**	**% N**	**C:N**	**Sample Type**	**Age (group)**	**Age (circa)**	**Sex**	**LEH (Y/N)**	**PO (Y/N)**	**CO (Y/N)**	**Skeletal Element**	**Source**
HUNG967	-14.9	13.0	6.18	42.0	15.1	2.9	Human	Infant I	2 to 3	N/A	N	N/A	N/A	rib	This study
HUNG968	-18.1	9.8	43.21	40.1	14.3	2.9	Human	Juvenile-Adult	16 to 22	M	N	N	N	cranium	This study
HUNG969	-17.5	11.6	1.05	25.6	9.0	3.2	Human	Adult-Mature	30 to 60	M	N	N	N	cranium	This study
HUNG177	-17.4	10.2	6.47	42.4	15.1	2.9	Human	Infant I-II	6 to 10	N/A	N	N	N	metacarpal	This study
HUNG175	-16.1	9.9	7.86	42.0	14.5	3.0	Human	Adult	20 to 39	F	N	N	N/A	metacarpal	This study
HUNG863	-17.9	9.9	4.00	39.1	14.0	3.0	Human	Adult	20 to 39	F	N	N	Y	cranium	This study
HUNG178	-20.0	9.9	6.88	42.0	14.9	3.0	Human	Adult	20 to 39	F	N	N	N	metatarsal	This study
HUNG401	-18.2	10.4	4.54	43.2	15.7	2.9	Human	Adult	20 to 39	M	N/A			rib	Gamarra *et al.*, 2018
HUNG417	-17.2	11.0	7.15	43.5	15.6	2.9	Human	Mature	40 to 59	F	N/A			rib	Gamarra *et al.*, 2018
HUNG418	-14.9	10.8	3.20	43.1	14.7	3.1	Human	Infant I	1 to 6	M	N/A			rib	Gamarra *et al.*, 2018
HUNG425	-17.2	10.9	6.05	43.3	15.7	2.9	Human	N/A			N/A			metacarpal	This study
HUNG82	-17.6	8.9	4.26	39.0	14.0	2.9	Human	Adult-Mature	20 to 59	N/A				ulna	This study
HUNG145	-17.9	10.0	13.35	44.5	16.3	2.9	Human	Adult	20 to 39	F	N	N	N/A	cranium	This study
HUNG146	-18.6	9.7	3.70	44.1	15.7	2.9	Human	Mature	40 to 59	F	Y	N	N	cranium	This study
HUNG148	-17.3	9.1	5.90	41.9	14.7	3.0	Human	Mature	40 to 59	F	N	N	N	metacarpal	This study
HUNG149	-16.8	9.5	5.87	42.8	15.2	2.9	Human	Mature	40 to 59	M	N	N	N	metatarsal	This study

Legend: F = female, M = male, N/A = not assessable; IA = Iron Age, BA = Bronze Age, EIA = Early Iron Age, EBA = Early Bronze Age, MBA = Middle Bronze Age, LBA = Late Bronze Age; LEH = linear enamel hypoplasia, PO = porotic hyperostosis, CO = cribra orbitalia, Y = Yes, N = No															
**ID**	**$\delta^{13}C$ ‰ (VPDB)**	**$\delta^{15}N$ ‰ (AIR)**	**% Colla-gen**	**% C**	**% N**	**C:N**	**Sample Type**	**Age (group)**	**Age (circa)**	**Sex**	**LEH (Y/N)**	**PO (Y/N)**	**CO (Y/N)**	**Skeletal Element**	**Source**
HUNG150	-16.7	10.0	8.26	43.6	15.7	2.9	Human	Mature	40 to 59	F	N	N	N/A	cranium	This study
HUNG151	-19.2	9.9	2.54	43.1	15.3	2.9	Human	Mature	40 to 59	F	N	N	N/A	cranium	This study
HUNG152	-16.9	10.5	11.12	43.9	15.8	2.9	Human	Adult	20 to 39	M	Y	N	N/A	cranium	This study
HUNG154	-17.6	9.0	21.16	44.8	16.2	2.9	Human	Adult-Mature	20 to 59	F	N	N	N/A	cranium	This study
HUNG155	-20.5	12.5	44.78	42.7	14.9	3.1	Human	Mature	40 to 59	M	Y	N	N	cranium	This study
HUNG156	-17.8	9.7	20.14	43.3	15.2	2.9	Human	Infant II	8 to 13	N/A	N	N	N/A	cranium	This study
HUNG158	-19.4	8.3	32.03	41.5	14.9	3.0	Human	Adult	20 to 39	F	N	N	N/A	metacarpal	This study
HUNG159	-15.6	10.7	4.25	44.6	15.9	3.0	Human	Juvenile	15 to 19	M	Y	N	N	cranium	This study
HUNG160	-17.6	10.6	4.00	42.7	15.2	2.9	Human	Adult-Mature	20 to 59	F	Y	N	Y	cranium	This study
HUNG161	-17.8	10.4	11.50	46.4	16.8	2.9	Human	Adult-Mature	20 to 59	M	Y	N	N	cranium	This study
HUNG162	-17.5	9.9	4.24	43.9	15.7	2.9	Human	Adult	20 to 39	F	N/A	N	N	cranium	This study
HUNG957	-17.3	9.2	2.85	42.7	14.9	3.1	Human	Adult	20 to 39	F	N/A			cranium	This study
HUNG958	-18.3	10.8	5.09	36.6	12.9	3.0	Human	Infant I	1 to 6	N/A	N/A			cranium	This study
HUNG960	-16.4	8.7	17.31	43.8	15.8	2.9	Human	Adult-Mature	20 to 59	M	N/A			cranium	This study

Supplementary Materials S3 – Statistical Results

BA = Bronze Age, IA = Iron Age, EBA = Early Bronze Age, MBA, = Middle Bronze Age, LBA = Late Bronze Age, EIA = Early Iron Age, LIA = Late Iron Age, F= Female, M = Male; SE = standard error, df = degrees of freedom, Sig. = Significance / p-value, S^2 = sum of squared differences, SD = standard deviation

**Tests of Normality**				
**Period**		**Shapiro-Wilk**		
		**Statistic**	**df**	**Sig.**
**$\delta^{13}C$**	BA	0.920	49	***0.003***
	IA	0.973	25	0.723
S3a				

**Tests of Normality**				
**Period**		**Shapiro-Wilk**		
		**Statistic**	**df**	**Sig.**
**$\delta^{15}N$**	BA	0.970	49	0.234
	IA	0.964	25	0.495
S3b				

**Tests of Normality**				
**Subperiods**		**Shapiro-Wilk**		
		**Statistic**	**df**	**Sig.**
**$\delta^{13}C$**	EBA	0.889	9	0.194
	MBA	0.841	18	***0.006***
	LBA	0.949	21	0.332
	EIA	0.978	24	0.855
S3c				

**Tests of Normality**				
**Subperiods**		**Shapiro-Wilk**		
		**Statistic**	**df**	**Sig.**
**$\delta^{15}N$**	EBA	0.945	9	0.632
	MBA	0.909	18	0.083
	LBA	0.915	21	0.069
	EIA	0.965	24	0.545
S3d				

**Mann-Whitney**				
**$\delta^{13}C$**	**U-value**	900.5	**p-value**	***0.001***
S3e				

**Levene Statistic**				
**$\delta^{13}C$**	**Statistic**	**df_1**	**df_2**	**Sig.**
	0.852	3	68	0.470
**Kruskal-Wallis**				
**$\delta^{13}C$**	**Statistic**	**df**	**p-value**	
	31.122	3	***<0.001***	
S3f				

Pairwise Mann-Whitney				
$\delta^{13}C$	**U-value**	EBA	MBA	LBA
	MBA	84		
	LBA	187	316.5	
	EIA	207	360	281
	**p-value**	EBA	MBA	LBA
	MBA	0.877		
	LBA	***<0.001***	***<0.001***	
	EIA	***<0.001***	***<0.001***	0.509
	**Bonferroni**	EBA	MBA	LBA
	MBA	1		
	LBA	***<0.001***	***0.001***	
	EIA	***<0.001***	***0.001***	1
S3g				

Independent t-test				
$\delta^{15}N$	**t-value**	**df**	**p-value**	IA
	3.369	72	BA	***0.001***
S3h				

Tukey's test				
$\delta^{15}N$	**p-value**	EBA	MBA	LBA
	MBA	1		
	LBA	0.858	0.716	
	EIA	0.266	0.128	***0.005***
S3i				

One-way ANOVA				
$\delta^{15}N$	$F_{(3, 68)}$	4.25	**p-value**	***0.008***
S3j				

Tests of Normality				
**Cultures**		**Shapiro-Wilk**		
		**Statistic**	**df**	**Sig.**
$\delta^{13}C$	Proto-Nagyrév	0.848	4	0.220
	Füzesabony	0.855	17	***0.013***
	Piliny or Piliny/Kyjatice	0.971	14	0.885
	pre-Gáva or Gáva	0.844	7	0.107
	pre-Scythian (Mezőcsát)	0.957	5	0.785
	Scythian (Vekerzug)	0.961	19	0.589
S3k				

Tests of Normality				
Cultures		Shapiro-Wilk		
		Statistic	df	Sig.
$\delta^{15}N$	Proto-Nagyrév	0.986	4	0.936
	Füzesabony	0.913	17	0.111
	Piliny or Piliny/Kyjatice	0.896	14	0.098
	pre-Gáva or Gáva	0.891	7	0.279
	pre-Scythian (Mezőcsát)	0.878	5	0.299
	Scythian (Vekerzug)	0.942	19	0.287
S3l				

Levene Statistic				
$\delta^{13}C$	**Statistic**	**df_1**	**df_2**	**Sig.**
	0.896	5	60	0.490
**Kruskal-Wallis**				
$\delta^{13}C$	**Statistic**	**df**	p-value	
	25.159	5	***<0.001***	
S3m				

Pairwise Mann-Whitney						
	**U-value**	Proto-Nagyrév	Füzesabony	Piliny or Piliny/Kyjatice	pre-Gáva or Gáva	pre-Scythian (Mezőcsát)
	Füzesabony	24				
	Piliny or Piliny/Kyjatice	54	190			
	pre-Gáva or Gáva	28	105.5	72		
	pre-Scythian (Mezőcsát)	20	78	57	19	
	Scythian (Vekerzug)	72	259	160	45	25
	**p-value**	Proto-Nagyrév	Füzesabony	Piliny or Piliny/Kyjatice	pre-Gáva or Gáva	pre-Scythian (Mezőcsát)
	Füzesabony	0.370				
	Piliny or Piliny/Kyjatice	0.006	0.005			
**$\delta^{13}C$**	pre-Gáva or Gáva	0.008	0.003	0.086		
	pre-Scythian (Mezőcsát)	0.014	0.005	0.042	0.808	
	Scythian (Vekerzug)	0.006	0.002	0.325	0.214	0.11
	**Bonferroni**	Proto-Nagyrév	Füzesabony	Piliny or Piliny/Kyjatice	pre-Gáva or Gáva	pre-Scythian (Mezőcsát)
	Füzesabony	0.300				
	Piliny or Piliny/Kyjatice	0.090	0.075			
	pre-Gáva or Gáva	0.120	***0.045***	1		
	pre-Scythian (Mezőcsát)	0.210	0.075	0.630	1	
	Scythian (Vekerzug)	0.090	***0.030***	1	1	1
S3n						

One-way ANOVA				
**$\delta^{15}N$**	**$F_{(5, 60)}$**	4.86	**p-value**	***0.001***
S3o				

Tukey's test						
	**p-value**	Proto-Nagyrév	Füzesabony	Piliny or Piliny/Kyjatice	pre-Gáva or Gáva	pre-Scythian (Mezőcsát)
**δ15N**	Füzesabony	0.403				
	Piliny or Piliny/Kyjatice	0.785	0.942			
	pre-Gáva or Gáva	0.673	1	0.998		
	pre-Scythian (Mezőcsát)	0.458	1	0.935	0.996	
	Scythian (Vekerzug)	***0.005***	***0.039***	***0.004***	0.114	0.535
S3p						

**Period/Subperiod**	**n**	**δ13C range (‰)**	**Mean**	**SD**	**δ15N range (‰)**	**Mean**	**SD**
Bronze Age Females	14	-20.25 to -17.21	-18.87	1.34	8.97 to 11.56	10.32	0.87
Iron Age Females	13	-19.4 to -16.10	-17.60	0.93	8.3 to 11.0	9.7	0.7
Bronze Age Males	21	-21.19 to -15.14	-18.71	1.71	9.36 to 12.91	11.16	0.85
Iron Age Males	8	-20.51 to -14.89	-17.14	1.73	8.73 to 12.49	10.45	1.09
S3q							

**Tests of Normality**				
**Females**		**Shapiro-Wilk**		
		**Statistic**	**df**	**Sig.**
**δ13C**	BA	0.776	13	***0.002***
	IA	0.927	12	0.308
S3r				

**Tests of Normality**				
**Females**		**Shapiro-Wilk**		
		**Statistic**	**df**	**Sig.**
**δ15N**	BA	0.916	13	0.194
	IA	0.944	12	0.504
S3s				

Tests of Normality				
**Males**		**Shapiro-Wilk**		
		**Statistic**	**df**	**Sig.**
**$\delta^{13}C$**	BA	0.944	20	0.265
	IA	0.949	7	0.792
S3t				

Tests of Normality				
**Males**		**Shapiro-Wilk**		
		**Statistic**	**df**	**Sig.**
**$\delta^{15}N$**	BA	0.955	20	0.422
	IA	0.449	7	0.449
S3u				

Mann-Whitney for Females by Period				
**$\delta^{13}C$**	**U-value**	IA	**p-value**	IA
	BA	42.5	BA	***0.019***
S3v				

Independent t-test for Males by Period				
**$\delta^{13}C$**	**t-value**	**df**	**p-value**	IA
	-2.216	27	BA	***0.035***
S3w				

Independent t-test for Females by Period				
**$\delta^{15}N$**	**t-value**	**df**	**p-value**	IA
	1.889	25	BA	0.070
S3x				

Independent t-test for Males by Period				
**$\delta^{15}N$**	**t-value**	**df**	**p-value**	IA
	1.867	27	BA	0.073
S3y				
